# Multisite chronic pain and the risk of autoimmune diseases: A Mendelian randomization study

**DOI:** 10.3389/fimmu.2023.1077088

**Published:** 2023-02-09

**Authors:** Yidan Tang, Weizhi Liu, Weishuang Kong, Shuangyi Zhang, Tao Zhu

**Affiliations:** ^1^ Department of Anesthesiology, West China Hospital, Sichuan University, Chengdu, China; ^2^ Laboratory of Anesthesia and Critical Care Medicine, National-Local Joint Engineering Research Centre of Translational Medicine of Anesthesiology, West China Hospital, Sichuan University, Chengdu, China; ^3^ Department of Surgery, Xuanwei Hospital of traditional Chinese Medicine, Xuanwei, China

**Keywords:** causal effect, autoimmune diseases, Mendelian, mediators, multisite chronic pain

## Abstract

**Background:**

Accumulating evidence has demonstrated that an association between chronic pain and autoimmune diseases (AIDs). Nevertheless, it is unclear whether these associations refer to a causal relationship. We used a two-sample Mendelian randomization (MR) method to determine the causal relationship between chronic pain and AIDs.

**Methods:**

We assessed genome-wide association study (GWAS) summary statistics for chronic pain [multisite chronic pain (MCP) and chronic widespread pain (CWP)], and eight common AIDs, namely, amyotrophic lateral sclerosis (ALS), celiac disease (CeD), inflammatory bowel disease (IBD), multiple sclerosis (MS), rheumatoid arthritis (RA), systemic lupus Erythematosus (SLE), type 1 diabetes (T1D) and psoriasis. Summary statistics data were from publicly available and relatively large-scale GWAS meta-analyses to date. The two-sample MR analyses were first performed to identify the causal effect of chronic pain on AIDs. The two-step MR and multivariable MR were used to determine if mediators (BMI and smoking) causally mediated any connection and to estimate the proportion of the association mediated by these factors combined.

**Results:**

With the utilization of MR analysis, multisite chronic pain was associated with a higher risk of MS [odds ratio (OR) = 1.59, 95% confidence interval (CI) = 1.01-2.49, *P* = 0.044] and RA (OR = 1.72, 95% CI = 1.06-2.77, *P* = 0.028). However, multisite chronic pain had no significant effect on ALS (OR = 1.26, 95% CI = 0.92-1.71, *P* = 0.150), CeD (OR = 0.24, 95% CI = 0.02-3.64, *P* = 0.303), IBD (OR = 0.46, 95% CI = 0.09-2.27, *P* = 0.338), SLE (OR = 1.78, 95% CI = 0.82-3.88, *P* = 0.144), T1D (OR = 1.15, 95% CI = 0.65-2.02, *P* = 0.627) or Psoriasis (OR = 1.59, 95% CI = 0.22-11.26, *P* = 0.644). We also found positive causal effects of MCP on BMI and causal effects of BMI on MS and RA. Moreover, there were no causal connections between genetically predicted chronic widespread pain and the risk of most types of AIDs disease.

**Conclusion:**

Our MR analysis implied a causal relationship between MCP and MS/RA, and the effect of MCP on MS and RA may be partially mediated by BMI.

## 1 Introduction

Chronic pain (CP), normally defined as the symptoms of ongoing pain lasting more than three months is a condition that has become a major global public health concern currently (1, 2). Around 30% of the global population suffers from chronic pain, with an average of three pain locations reported (3). Multisite chronic pain (MCP) and chronic widespread pain (CWP), as a derived chronic pain phenotype, are widely spread among the older population (4, 5). The global prevalence of CWP in the general population ranged from 10.6% to 11.8% (6). In fact, The number of chronic pain sites, generally three, is currently regarded as a strong prognostic indicator for continued chronic pain courses (7). The previous study also demonstrated that patients with CP, particularly those with obesity or inactivity, increases the risk of their offspring developing additional chronic pain sites (8). Moreover, CP is associated with expensive health care costs, rising disability and mortality (9–13).

Autoimmune diseases (AIDs) are a set of complex chronic illnesses with unknown etiology that are characterized by a lack of autoimmune tolerance (14). In fact, approximately 5%–8% of the world population suffers from these diseases (15). Some studies suggest that the prevalence of AIDs have been steadily increasing over the previous few decades (16). The cost of treating individuals with AID is likely to be substantial in terms of both direct and indirect expenses (17, 18). Besides, AIDs are regarded as major sources of illness and death globally (16).

The relationship between CP and AIDs has been studied to some extent, yet the results remain uncertain. In rheumatoid arthritis (RA), the frequent disconnect between pain and inflammation is an unexplained aspect of chronic pain. Joint pain often precedes evidence of joint inflammation and is therefore also considered to be one of the early signs of emerging RA (5). Moreover, duo to pain connected to developing psoriasis, long-term acetaminophen and NSAIDs use may be associated with an increased risk of psoriasis (19). Fibromyalgia, one of CWP, seems to contribute to constitutional symptoms more in Systemic Lupus Erythematosus (SLE) (20). The causal association between CP and AIDs, on the other hand, has yet to be demonstrated, which is incredibly essential and might improve our current understanding of their pathogeneses.

Recent prospective studies have found that people suffering from chronic pain are more likely to be overweight (as assessed by body mass index [BMI]), and to smoke (21, 22), despite the fact that all of these are well-known risk factors for AIDs (23, 24). As a result, obesity and smoking appear to be possible mediators of chronic pain and AIDs. Given that existing chronic pain therapy is often inadequate, determining if these hypothesized mediators are causative and, if so, establishing intervention techniques that target them might assist to lower AIDs risk associated with chronic pain from both a public health and clinical standpoint.

Observational researches are susceptible to confounding factors such as demographics or environmental exposure. Mendelian randomization (MR) is an alternative method of determining the causal association between an exposure and a disease result by using genetic variations as environmental exposure proxies (25). Because genetic variations are thought to be assigned at random before birth, they are relatively independent of environmental influences and established well before illness onset, limiting residual confounding and reverse causation difficulties that restrict traditional observational researches (26). In the present study, we applied a two-sample MR framework to explore whether MCP and CWP contribute to the development of eight major AIDs including amyotrophic lateral sclerosis (ALS), celiac disease (CeD), inflammatory bowel disease (IBD), multiple sclerosis (MS), RA, SLE, type 1 diabetes (T1D) and psoriasis. MR mediation analyses were further implemented to test whether BMI and smoking initiation mediate any relationship. We hypothesized that MCP would increase the risk of AIDs potentially through mediated effect of BMI.

## 2 Materials and methods

**Figure 1:**
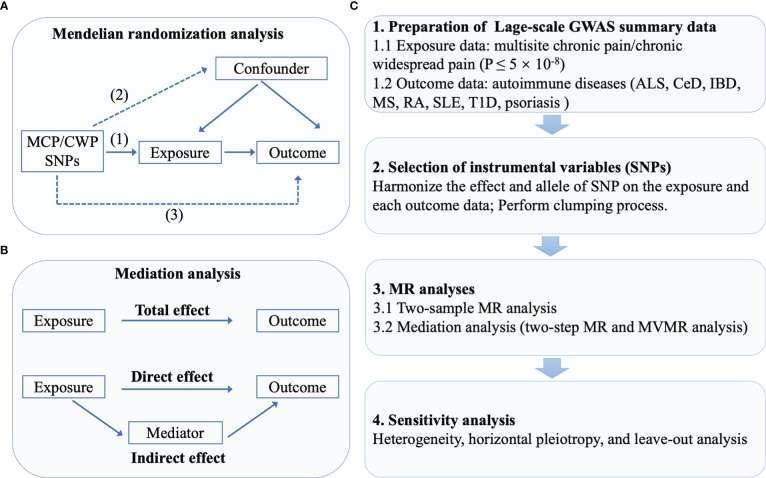
The flowchart of the study. **(A)** The principles for mendelian randomization study are as follows: (1) genetic instrumental variables are associated with the exposure, (2) genetic instrumental variables are independent of confounding variables, (3) genetic instrumental variables only affect outcome through exposure. **(B)** In mediation analysis, the total effect was decomposed into direct effect and indirect effect (derived by subtracting direct effect from total effect). **(C)** The whole workflow of MR analysis.

We obtained the research summary dataset from publicly published studies approved in respective studies by the institution themselves. Due to the fact that all the data utilized was already in the public domain, there is no additional approval required. A two-sample MR was utilized to assess the causal association between CP and AIDs, including ALS, CeD, IBD, MS, RA, SLE, T1D, and psoriasis (23–25). Briefly, MCP and CWP served as the exposures, while eight subtypes of AIDs served as the outcomes. As instrumental variables (IVs), single-nucleotide polymorphisms (SNPs) strongly associated with MCP and CWP were selected. In MR, 3 crucial assumptions for the genetic IVs to be valid instruments are as follows: (1) genetic instrumental variables are associated with the exposure, (2) genetic instrumental variables are independent of confounding variables, (3) genetic instrumental variables only affect outcome through exposure (27). A series of sensitivity analyses for significant associations were conducted. The flowchart is shown in **Figures 1A, C**.

### 2.1 MCP and CWP samples

Summary statistics for MCP and CWP were sourced from a large-scale biomedical database of GWAS: the UK Biobank Consortium. SNPs associated with MCP were identified in a sample of 387,649 people from the Johnston K et al. study (28). SNPs associated with CWP were identified from Rahman et al. study, which comprised 6,914 cases and 242,929 controls (29). MCP is a quantitative phenotype defined as the total of self-reported pain lasting at least three months in seven separate bodily regions (head, face, neck/shoulder, back, stomach/abdomen, hip, and knee) (28). During the baseline investigations, UK Biobank participants were asked *via* a touchscreen questionnaire about “pain types experienced in the last month”, with possible answers: ‘None of the above’; ‘Prefer not to answer’; pain at seven different body sites (head, face, neck/shoulder, back, stomach/abdomen, hip, knee); or ‘all over the body’. The seven individual body-site pain options were not mutually exclusive and participants could choose as many as they felt appropriate. As a result, the scores for multisite chronic pain ranged from 0 to 7. In addition, CWP is described as a combination of self-reported diagnosis of pain all over the body lasting more than 3 months, simultaneous pain in the knee, shoulder, hip, and back lasting more than 3 months, and fibromyalgia (29). Details of GWAS studies are provided in the **Table 1**.

**Table 1:** Details of GWAS studies for pain-related traits.

Trait	Sample sizes (Case/Control)	R^2^	F-statistic	Consortium/Reference
Multisite chronic pain (MCP)	387,649	0.0042	42	UK Biobank (33830993)
Chronic widespread pain (CWP)	6,914/242,929	0.0133	31	UK Biobank (33926923)

### 2.2 AIDs samples and mediators

The two-sample MR approach extracts genetic instruments for exposure and outcome from separate datasets, which increases statistical power, but additional sources of bias may be introduced if the two samples used in a study overlap (30). Here, to avoid potential bias associated with sample overlap, we selected GWAS meta-analyses for eight AID diseases and excluded the UK Biobank cohort, as chronic pain data consisted of UK Biobank only. Besides, to eliminate population stratification bias, all SNPs and corresponding summary data were retrieved from studies that solely included European ancestry. AIDs genetic data was gathered from eight independent GWAS for each disease. Summary statistics for ALS were derived from the Nicolas et al. study, which comprised 20,806 cases and 59,804 controls (31). Summary statistics of CeD were derived from the Trynka et al. study, including 11,812 cases and 229 controls (32). Summary statistics of IBD were derived from the Liu et al. study, including 31,665 cases and 33,977 controls (33). Summary statistics of MS were derived from the latest GWAS meta-analysis of the International MS Genetics Consortium (IMSGC), including 47,429 cases and 68,374 controls (34). Summary statistics of RA were derived from the Okada et al. study, including 14,361 cases and 43,923 controls (35). Summary statistics of SLE were derived from the Bentham et al. study, including 5,201 cases and 9,066 controls (36). Summary statistics of T1D were derived from the Forgetta et al. study, including 9,266 cases and 15,574 controls (37). Finally, summary statistics of Psoriasis were derived from Tsoi et al. study, including 10,588 cases and 22,806 controls (38). All included GWAS meta-analyses are publicly available, and the sample size is essentially the greatest to yet, ensuring the strength of instruments. In this MR analysis, we identified BMI and smoking initiation as potential intermediate risk variables based on the available literature and GWAS summary data. Details of GWAS studies are provided in the **Table 2**.

**Table 2:** Details of GWAS studies for AIDs traits and Mediators.

	Trait	Sample sizes (Case/Control)	Consortium/Reference
**Outcomes**	ALS	20,806/59,804	Nicolas et al. (29566793)
	CeD	11,812/229	Trynka et al. (22057235)
	IBD	31,665/33,977	Liu et al. (26192919)
	MS	47,429/68,374	IMSGC (31604244)
	RA	14,361/43,923	Okada et al. (24390342)
	SLE	5,201/9,066	Bentham et al. (26502338)
	T1D	9,266/15,574	Forgetta et al. (32005708)
	Psoriasis	10,588/22,806	Tsoi et al. (23143594)
**Mediators**	BMI	152,893	Locke et al. (25673413)
	Smoking initiation	311,629/321,173	Liu et al. (30643251)

ALS, amyotrophic lateral sclerosis; CeD, celiac disease; IBD, inflammatory bowel disease; MS, multiple sclerosis; RA, rheumatoid arthritis; SLE, systemic lupus erythematosus; T1D, type 1 diabetes; BMI, body mass index.

### 2.3 Genetic instruments selection and harmonization

To identify suitable instrumental variables (IVs), a variety of quality control procedures were conducted. Firstly, independent SNPs linked with MCP and CWP at genome-wide significance *(P*-value < 5×10^-8^) were selected as potential IVs. Secondly, a clumping strategy with a threshold of *r^2^* < 0.001 and kb = 10,000 was used to reduce linkage disequilibrium (LD). Finally, to evaluate the strength of the selected SNPs, the F statistics were calculated using the formula F = R^2^ (N–k–1)/[(1–R^2^) k], where R^2^ is the proportion of variability explained by each SNP, N is the sample size of the GWAS, and k is the number of SNPs. When the F-statistic is <10, it indicates that IV is a weak instrument (39).

### 2.4 Two-step MR and mediation analysis

Two-step MR was employed to determine if an intermediate attribute acts as a mediator between exposure and outcome (40). The first step was to calculate the causal effect of MCP on putative mediators. In the second step, SNPs for potential mediating risk variables were utilized to genetically predict these mediators and assess their causal effect on the AIDs. For MR mediation analysis, potential mediators which presented supporting evidence for two stages in MR (causal effects of MCP on mediators and causal effects of mediators on AIDs) were considered (5). The entire effect of MCP on AIDs as assessed by MR can be split into direct (not mediated by mediators) and indirect effects (effect mediated by the mediators). Therefore, body mass index [BMI, (SD, ~4.8 kg/m^2^)] and smoking initiation were considered as the potential mediators (41, 42). The indirect effect of MCP on MS (or RA), through BMI, was obtained by multiplying the effect of MCP on BMI and the effect of BMI on MS. The same process applied to mediation analysis of smoking. Notably, MR mediation analysis necessitates that mediator be continuous variable in order to eliminate any bias in the estimation of mediating effects (27). We therefore only measured the proportion of MCP’s effect that was mediated by BMI. **Figure 1B** exhibited a brief description of mediation analysis.

### 2.5 Statistical analysis

For univariable MR analysis, a two-sample MR analysis was utilized to evaluate whether there is a causal relationship between MCP/CWP on AIDs. As a genetic method, MR enables the evaluation of inferences about causal exposure-outcome relationships by eliminating reverse causality and minimizing the risk of confounding (27). When more than two SNPs are identified, the inverse variance weighted model (IVW) with fixed effect is used to aggregate MCP causation for each AIDs; when only one SNP is detected, the Wald ratio is applied for MR analysis. Unless all genetic differences in the investigation are legitimate instrumental factors, estimates will be inconsistent. Moreover, MR-Egger, simple mode, weighted median, weighted mode techniques were severed as sensitivity analysis methods. Under a weaker assumption, the MR-Egger technique may determine if genetic variations have pleiotropic effects on the result that deviate on average from zero (directional pleiotropy) and offer a consistent estimate of the causative influence (43). Horizontal pleiotropy is described as some instruments additionally influence the outcome through pathways that bypass the exposure (44). Besides, the MR pleiotropy residual sum and outlier (MR-PRESSO) test was also applied to identify possible horizontal pleiotropy and eliminate pleiotropy impacts by removing outliers (30). The leave-one-out analysis was conducted to investigate whether the significant results were influenced by a particular SNP. For multivariable MR (MVMR) analysis, we employed the inverse-variance weighted method, with MR-Egger serving as sensitivity analysis. Cochran’s Q-statistic was used to the heterogeneity.

A *P*-value of less than 0.05 was selected as the statistical significance threshold. To adjust for multiple testing (multiple exposures), the statistical significance of the MR effect estimates was defined at a false discovery rate (FDR) of less than 5% (45, 46). All analyses were carried out using packages “TwoSampleMR”, “MendelianRandomization”, “MRPRESSO” and “fdrtool” in R version 4.2.1.

## 3 Results

### 3.1 Selection of instrumental variables

After a series of control steps for quality test, 39 SNPs and 3 SNPs related with MCP and CWP, respectively, were identified as IVs. The F statistics of all identified SNPs were more than 10 (**Table 1**). Specifically, 38 SNPs independent SNPs were associated with MCP for ALS, 4 independent SNPs were associated with MCP for CeD, 4 independent SNPs were associated with MCP for IBD, 32 independent SNPs were associated with MCP for MS, 30 independent SNPs were associated with MCP for RA, 34 independent SNPs were associated with MCP for SLE, 38 independent SNPs were associated with MCP for T1D, 4 independent SNPs were associated with MCP for Psoriasis. The harmonies data for MCP and AIDs was shown in **Supplemental Table S1**.

3 SNPs independent SNPs were associated with CWP for ALS, no independent SNPs were associated with CWP for CeD, 1 independent SNPs were associated with CWP for IBD, 2 independent SNPs were associated with CWP for MS, 2 independent SNPs were associated with CWP for RA, 2 independent SNPs were associated with CWP for SLE, 3 independent SNPs were associated with CWP for T1D, no independent SNPs were associated with CWP for Psoriasis. The harmonies data for CWP and AIDs was shown in **Supplemental Table S2**. The selected IVs for mediation analysis was shown in **Supplemental Tables S7**-**S10**.

The F-statistics of IVs are more than 10, indicating no evidence of weak instrument bias.

### 3.2 Causal effects of MCP on AIDs, BMI and smoking behaviors

**Figure 2:**
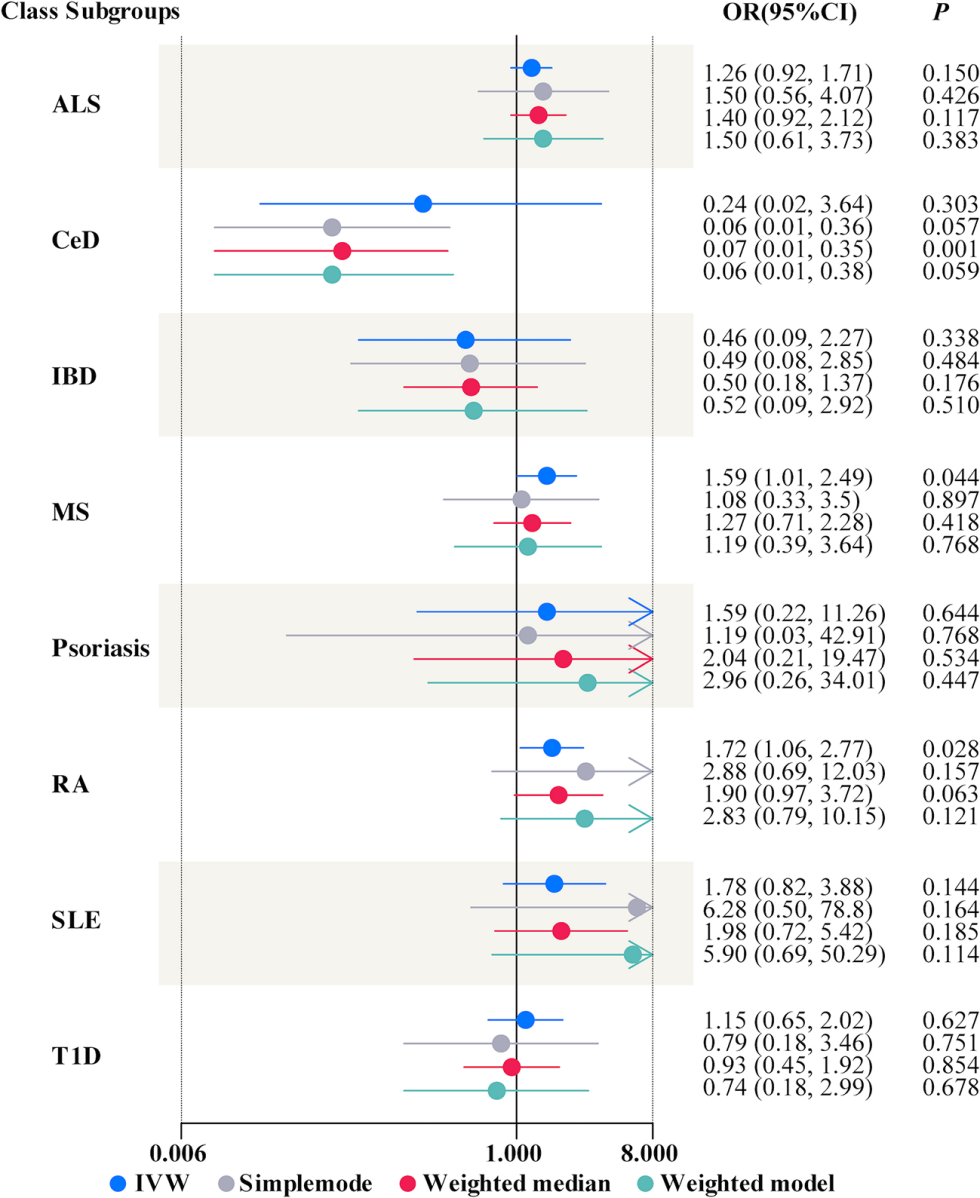
Forest plots summarizing the causal effects of multisite chronic pain on autoimmune diseases. OR, odds ratio; CI, confidence interval; ALS, amyotrophic lateral sclerosis; CeD, celiac disease; IBD, inflammatory bowel disease; MS, multiple sclerosis; RA, rheumatoid arthritis; SLE, systemic lupus erythematosus; T1D, Type 1 diabetes; IVW, inverse variance weighted.

In univariable MR, a higher genetically predicted MCP was associated with a higher risk of MS (IVW: OR = 1.59, 95% CI = 1.01-2.49, *P* = 0.044, FDR-corrected *P* < 0.05) and RA (IVW: OR = 1.72, 95% CI = 1.06-2.77, *P* = 0.028, FDR-corrected *P* < 0.05) (**Figure 2**). Besides, MCP was no significant effect on ALS (IVW: OR = 1.26, 95% CI = 0.92-1.71, *P* = 0.150), CeD (IVW: OR = 0.24, 95% CI = 0.02-3.64, P = 0.303), IBD (IVW: OR = 0.46, 95% CI = 0.09-2.27, *P* = 0.338), SLE (IVW: OR = 1.78, 95% CI = 0.82-3.88, *P* = 0.144), T1D (IVW: OR = 1.15, 95% CI = 0.65-2.02, *P* = 0.627) or Psoriasis (IVW: OR = 1.59, 95% CI = 0.22-11.26, *P* = 0.644) (**Figure 2** and **Supplemental Table S3**). In addition, the results of MR-Egger, the Weighted Median, Weighted Mode and simple mode were shown in **Figure 2** and **Supplemental Table S3**. Moreover, we found evidence that genetic liability to MCP led to higher BMI (IVW: OR = 1.19, 95% CI = 1.26-1.67, *P* = 0.027) and increased smoking initiation (IVW: OR = 1.43, 95% CI = 1.19-1.73, *P* < 0.001) (**Table 3**).

**Table 3:** Causal effect of multisite chronic pain on mediators and of mediators on MS/RA in two-step Mendelian randomization analyses.

Mediators	Method	Effect of MCP on mediators		Effect of mediators on MS		Effect of mediators on RA	
		Effect estimate: OR (95% CI)	*P*	Effect estimate: OR (95% CI)	*P*	Effect estimate: OR (95% CI)	*P*
BMI	IVW	1.19 (1.26, 1.67)	0.027	1.28 (0.99, 1.66)	0.057	1.43 (1.07, 1.92)	0.016
	MR Egger	1.59 (0.53, 4.71)	0.414	1.56 (0.79, 3.09)	0.214	0.55 (0.26, 1.16)	0.130
Smoking initiation	IVW	1.43 (1.19, 1.73)	0.0001	1.04 (0.88, 1.23)	0.642	1.50 (1.17, 1.92)	0.001
	MR Egger	0.75 (0.33, 1.70)	0.493	0.67 (0.28, 1.59)	0.411	1.32 (0.39, 4.49)	0.655

MCP, multisite chronic pain; MS, multiple sclerosis; RA, rheumatoid arthritis; OR, odds ratio; CI, confidence interval; BMI, body mass index; IVW, inverse-variance weighted.

### 3.3 Causal effects of BMI and smoking behaviors on MS/RA

In univariable MR, there was no significant effect for BMI (IVW: OR = 1.28, 95% CI = 0.99-1.66, *P* = 0.057, FDR-corrected *P* = 0.435) or smoking initiation (IVW: OR = 1.04, 95% CI = 0.88-1.23, *P* = 0.642, FDR-corrected *P* = 0.897) on MS (**Table 3**; **Supplemental Table S11**). Besides, higher BMI (IVW: OR = 1.43, 95% CI = 1.07-1.92, *P* = 0.016, FDR-corrected *P* < 0.05) or increased smoking initiation exposure (IVW: OR = 1.50, 95% CI = 1.17-1.92, *P* = 0.001, FDR-corrected *P* < 0.05) led to a higher risk of RA (**Table 3**; **Supplemental Table S11**).

### 3.4 Mediation by BMI and smoking behaviors

In the MVMR analysis of MCP-BMI-MS, the direct effect of MCP on MS was OR 1.81 (95% CI = 1.11-2.94, *P* = 0.017) after accounting for BMI, and the direct effect of BMI on MS was OR 0.59 (95% CI = 0.19-1.88, *P* = 0.379) after accounting for MCP (**Table 4**; **Supplemental Table S18**). The proportion mediated by BMI was 9%. For the MVMR analysis of MCP-smoking-MS, the direct effect of MCP on MS was OR 1.45 (95% CI = 0.80-2.63, *P* = 0.220) after accounting for smoking, and the direct effect of smoking on MS was OR 1.15 (95% CI = 0.49-2.71, *P* = 0.747) after accounting for MCP (**Table 4**; **Supplemental Table S18**).

**Table 4:** Causal effect of multisite chronic pain on MS/RA in multivariable Mendelian randomization analyses.

	Effect of MCP on MS		Effect of MCP on RA	
Mediators adjusted	Effect estimate (IVW): OR (95% CI)	*P*	Effect estimate (IVW): OR (95% CI)	*P*
BMI	1.81 (1.11, 2.94)	0.017	2.21 (1.05, 4.68)	0.037
Smoking initiation	1.45 (0.80, 2.63)	0.220	1.48 (0.79, 2.78)	0.219

Abbreviations: MCP, multisite chronic pain; MS, multiple sclerosis; RA, rheumatoid arthritis; OR, odds ratio; CI, confidence interval; BMI, body mass index.

In the MVMR analysis of MCP-BMI-RA, the direct effect of MCP on RA was OR 2.21 (95% CI = 1.05-4.68, *P* = 0.037) after accounting for BMI and the direct effect of BMI on RA was OR 0.52 (95% CI = 0.11-2.38, *P* = 0.398) after accounting for MCP (**Table 4**; **Supplemental Table S18**). The proportion mediated by BMI was 11%. For the MVMR analysis of MCP-smoking-RA, the direct effect of MCP on RA was OR 1.48 (95% CI = 0.79-2.78, *P* = 0.219) after accounting for smoking, and the direct effect of smoking on RA was OR 1.43 (95% CI = 0.53-3.80, *P* = 0.479) after accounting for MCP (**Table 4**; **Supplemental Table S18**).

### 3.5 Sensitivity analysis

**Figure 3:**
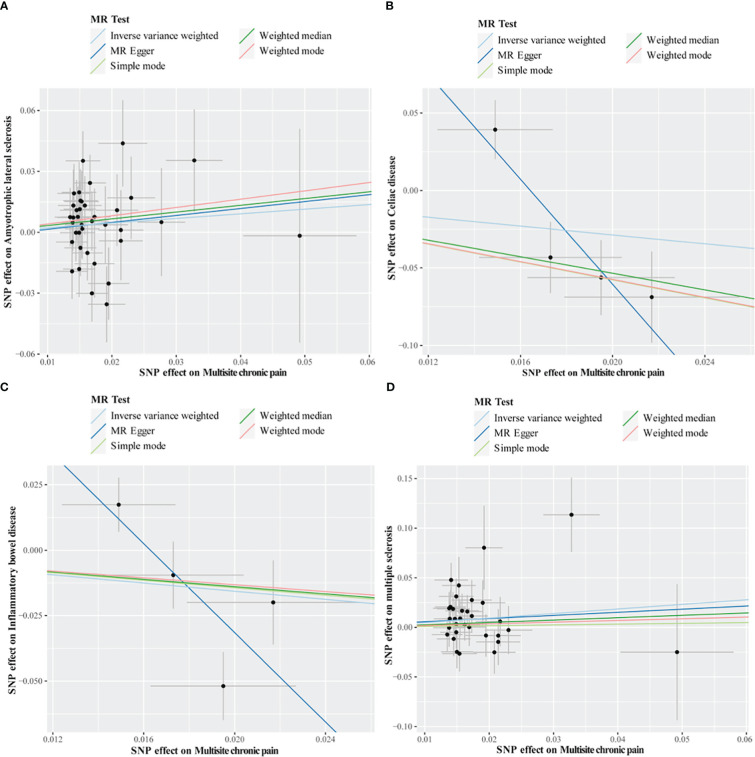
Scatter plots of MR analysis. The slope of each line corresponding to the estimated MR effect based on various models. **(A)** multisite chronic pain on amyotrophic lateral sclerosis, **(B)** multisite chronic pain on celiac disease, **(C)** multisite chronic pain on inflammatory bowel disease, **(D)** multisite chronic pain on multiple sclerosis.

**Figure 4:**
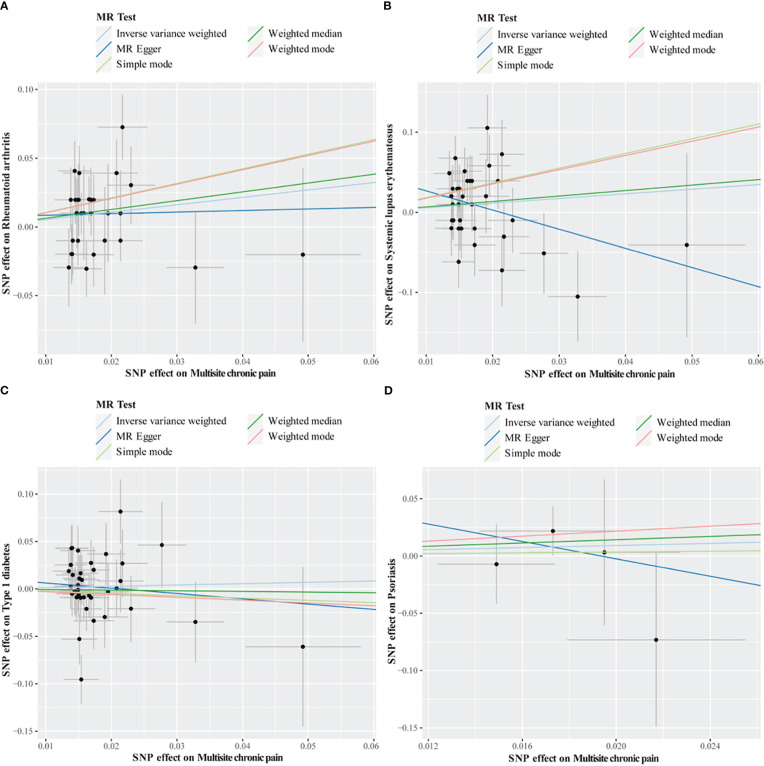
Scatter plots of MR analysis. The slope of each line corresponding to the estimated MR effect based on various models. **(A)** multisite chronic pain on rheumatoid arthritis, **(B)** multisite chronic pain on systemic lupus erythematosus, **(C)** multisite chronic pain on type 1 diabetes, **(D)** multisite chronic pain on psoriasis.

Cochran’s Q test revealed no heterogeneity for MCP IVs in most AIDs types (ALS, MS, RA, SLE, T1D, and Psoriasis), but considerable heterogeneity for CeD (Q = 13.76; *P* = 0.003) and IBD (Q = 15.93; *P* = 0.001) (**Table 5**; **Supplemental Table S4**). For the MR analysis of the MCP on AIDs, none of the MR-Egger regression intercepts deviated from the null value, indicating that horizontal pleiotropy was not present (all intercept P-values were greater than 0.05) (**Table 5**; **Supplemental Table S5**). Additionally, the MR-PRESSO test identified one (rs11871043) and two (rs11871043, rs7628207) outliers for CeD and IBD, respectively. After removal of outliers, the association was significant for MCP on CeD (outlier-corrected IVW: OR = 0.06; 95% CI = 0.01-0.25; *P* = 0.0002), but no significant effect on MCP for IBD (outlier-corrected IVW: OR = 0.48; 95% CI = 0.17-1.34; *P* = 0.16) (**Table 5**; **Supplemental Table S6**). The scatter plots of causal association of MR analysis are shown in **Figures 3**, **4**. Leave-one-out analysis indicated that the causal effects were not driven by any single SNP (**Supplemental Figures S1**, **S2**).

**Table 5:** Associations between genetically instrumented Multisite chronic pain and autoimmune diseases status biomarkers.

Exposure	Outcome	N SNPs	IVW Heterogeneity test		MR-Egger pleiotropy test			MR-PRESSO global outlier test
			Q	*P*	intercept	SE	*P*	Outlier
MCP	ALS	38	39.5	0.36	-0.002	0.01	0.88	None
	CeD	4	13.8	0.003	0.28	0.09	0.10	rs11871043
	IBD	4	15.9	0.001	0.14	0.08	0.23	rs11871043, rs7628207
	MS	32	37.9	0.18	0.002	0.02	0.90	None
	RA	30	31.9	0.32	0.007	0.02	0.71	None
	SLE	34	45.8	0.07	0.05	0.03	0.10	None
	T1D	38	44.7	0.18	0.01	0.02	0.61	None
	Psoriasis	4	1.79	0.62	0.07	0.18	0.72	None

IVW, inverse-variance weighted; MR-PRESSO, MR-Pleiotropy Residual Sum and Outlier method; SE, standard error of coefficient estimate; MCP, multisite chronic pain; ALS, amyotrophic lateral sclerosis; CeD, celiac disease; IBD, inflammatory bowel disease; MS, multiple sclerosis; RA, rheumatoid arthritis; SLE, systemic lupus erythematosus; T1D, type 1 diabetes.

We observed some heterogeneity in MR analyses for the effect of MCP on mediators (BMI and smoking initiation) and the effect of mediators on MS/RA indicated by Cochran’s Q statistic (**Supplemental Table S12**). The results of pleiotropy test for MCP on mediators and mediators on MS/RA were **Supplemental Table S13**. Leave-one-out analysis indicated that no individual SNP was observed to drive the overall results (**Supplemental Tables S14**-**S17**). In the MVMR analysis, Cochran’s Q test revealed no heterogeneity and the estimates by MR egger method were shown in **Supplemental Table S19**.

### 3.6 Causal effects of CWP on AIDs

Overall, there were no causal connections between genetically predicted CWP and the risk of most types of AIDs disease, although CWP had a significant effect on IBD risk (Wald ratio; P = 0.0002) (**Supplementary Table S20**). Furthermore, the MR-Egger, Weighted Median, and Weighted Mode methods produced consistent results for CWP on ALS and T1D, respectively. No evidence of heterogeneity was observed between the genetic IVs for CWP (**Supplementary Table S21**). No directional pleiotropy for CWP on T1D was found by MR-Egger regression analysis (intercept = -0.14; se = 0.12; P = 0.45), but there was directional pleiotropy for CWP on ALS (intercept = -0.11; se = 0.04; P = 0.01) (**Supplementary Table S22**). Not enough IVs was available for the MR-PRESSO global test.

## 4 Discussion

To the best of our knowledge, this is the first large-scale two-sample MR study to investigate the potential causative relationship between MCP and AIDs. In this current study, a large-scale biomedical database from UK Biobank was utilized to identify MCP and CWP, and whether there is a casual effect on eight subtypes of AIDs. Our study has sufficient power to detect a small effect of chronic pain within the MR framework by using 39 lead SNPs associated with MCP as genetic instruments. Our results reveal that MCP is causally associated with higher risk of MS and RA, and the effect of MCP on RA may be partially mediated by BMI. Our findings further highlighted that BMI play important role in causally mediating the effect of MCP on MS/RA. In addition, we did not perform MR analysis of the effect of AIDs on MCP/CWP because there were no SNP loci available for reverse MR.

A study reported that pain induces relapses of autoimmune encephalomyelitis (MS model) in mice, possibly related to specific sensory-sympathetic signaling triggered by pain induction (47). Our results are consistent with this, and MCP maybe associated with an increased risk of MS (OR = 1.59, *P* = 0.044). In addition, a previous study has indicated that joint pain) usually precedes signs of joint inflammation and is one of the first indicators of new-onset RA (34). This supports our findings and suggests that MCP is a potential risk factor for RA (OR = 1.72, *P* = 0.028). In addition, there were no significant differences between MCP/CWP and other AIDs types (ALS, CeD, IBD, SLE, T1D, and Psoriasis). Interestingly, a Taiwanese nationwide population-based study found that patients with interstitial cystitis/bladder Pain had a higher prevalence of several AIDs, including RA, SLE, Sjögren syndrome (SS), ankylosing spondylitis (AS), and IBS (35).

Our results are consistent with earlier MR studies that have shown positive causal effects of BMI and smoking on RA/MS, and positive causal effects of MCP on BMI and smoking initiation (5, 48–50). Together, these findings provided support for BMI and smoking as causative mediators (mechanisms) linking a larger number of chronic pain sites to an increased risk of developing MS/RA. In further multivariable MR analysis, the effect sizes for MCP on RA were attenuate with adjustment for BMI and smoking initiation. Smoking is considered to be a risk factor for MS, and a pooled analysis of several small studies showed an OR of ∼1.5 for smoking and a dose-response relationship between smoking and MS risk: cumulative smoking was associated with increased risk (51). However, no significant differences were detected in our univariable MR or MVMR analysis, which may be related to sample size, ethnicity, etc.

Our findings may have potential public health and clinical practice consequences. Given the accumulating evidence that multisite chronic pain is a causative factor for MS/RA, the effective therapy of individuals with chronic pain, especially those with multi-site chronic pain, may be advantageous for preventing MS/RA. Some people reported chronic pain that persisted for a long time even after treatment (52). Therefore, effective interventions targeting downstream mediators of chronic pain (such as BMI and smoking) would give a chance to minimize the risk of MS/RA in patients with untreated chronic pain.

In addition, there are some limitations in this study. Firstly, all the participants’ data in this study were collected from European country to avoid potential bias from ethnic difference. Nevertheless, the conclusions of other region people on the incidental link between MCP/CWP and AIDs remained unknown. Secondly, in the MVMR analysis, we only assessed the fraction of multi-site chronic pain mediated by BMI due to the non-overlapping character of OR values. Future approaches should be developed to address the bias of binary exposure or mediation in MR analysis in order to appropriately evaluate mediating effects. Thirdly, due to the use of public databases and the lack of demographic data (e.g., gender and ethnicity) in the original study, additional subgroup analyses were not possible. Furthermore, we were unable to investigate exposure-mediated interactions because estimates would be biased if BMI interacted with MCP’s causal influence on RA/MS risk. The current analysis assumes that MCP has a linear effect on each outcome; nevertheless, even if this assumption is invalid, the current analysis is a valid test of the causal null hypothesis.

In conclusion, our results demonstrated genetic evidence of a potential causal relationship between MCP and MS or RA in the European population, which may partially mediate through BMI. Further study is required to elucidate the pathophysiology of the causal relationship between MCP and MS/RA.

## Data availability statement

The original contributions presented in the study are included in the article/**Supplementary Material**. Further inquiries can be directed to the corresponding authors.

## Author contributions

YT and TZ designed the study. YT, WK and SZ collected and analyzed the data. YT, WL, WK, SZ and TZ wrote the manuscript. All authors contributed to the article and approved the submitted version.
