# Supplementary material for: Multisite chronic pain and the risk of autoimmune diseases: A Mendelian randomization study
Source: Front Immunol. 2023 Feb 9;14:1077088. doi: 10.3389/fimmu.2023.1077088 (PMC9947645; doi:10.3389/fimmu.2023.1077088)
Supplement: Supplementary file 1 [file DataSheet_1.docx]

Supplementary Material

# Supplementary Figures

## Supplementary Figure S1


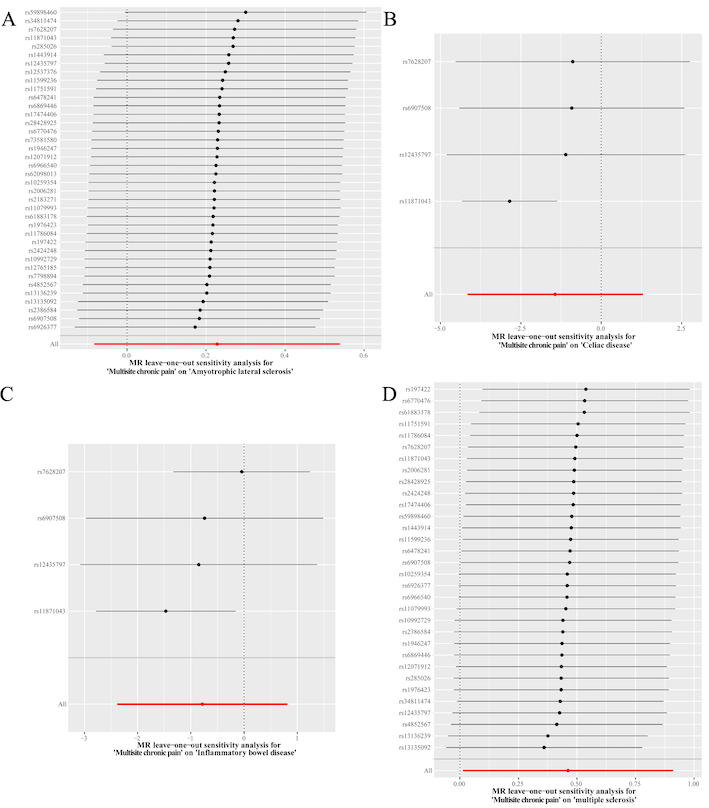


**Supplementary Figure S1.** Forest plots of leave-one-out for MCP on Amyotrophic lateral sclerosis (**A**), Celiac disease (**B**), Inflammatory bowel disease (**C**), and Multiple sclerosis (**D**).

## Supplementary Figure S2


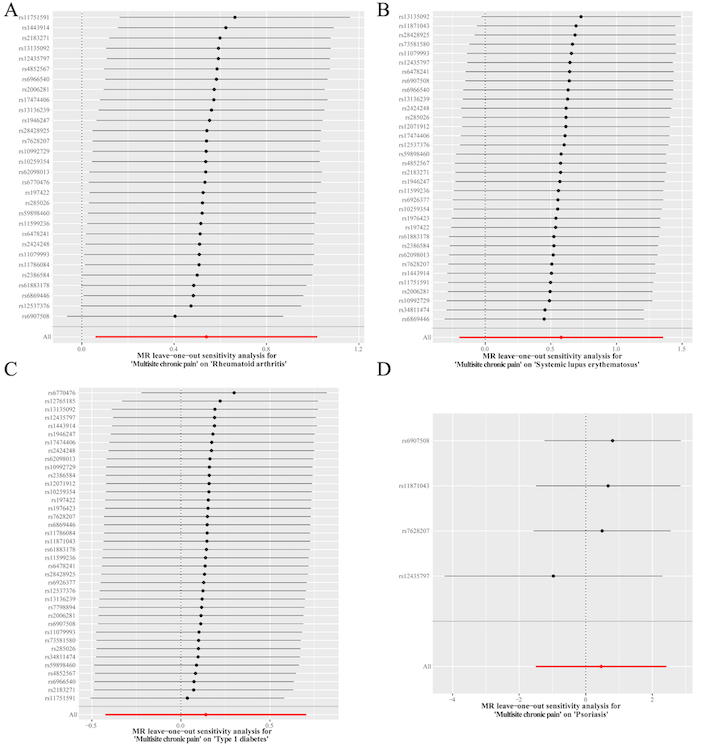


**Supplementary Figure S2.** Forest plots of leave-one-out for MCP on Rheumatoid arthritis (**A**), Systemic lupus erythematosus (**B**), Type 1 diabetes (**C**), and Psoriasis (**D**).

# Supplementary Tables

## Supplementary Table S1 Harmonies data for MCP and AIDs.

| SNP | **EA** | **OA** | **Exposure (MCP)** | | | **Outcome (AIDs)** | | | |
| --- | --- | --- | --- | --- | --- | --- | --- | --- | --- |
|  |  |  | **beta** | **se** | ***P*-value** | **Outcome** | **beta** | **se** | ***P*-value** |
| rs10259354 | G | A | 0.0147 | 0.0026 | 3.00E-08 | SLE | 0.0295588 | 0.0365629 | 0.418839 |
| rs10888692 | C | G | -0.014 | 0.0025 | 5.30E-09 | SLE | -0.0392207 | 0.0308228 | 0.203211 |
| rs10992729 | C | T | 0.0158 | 0.0026 | 1.10E-09 | SLE | 0.0512933 | 0.029611 | 0.0832319 |
| rs11079993 | G | T | -0.017 | 0.0025 | 5.70E-12 | SLE | 0.0202027 | 0.0281221 | 0.472517 |
| rs11599236 | T | C | 0.0138 | 0.0025 | 3.30E-08 | SLE | 0.0202027 | 0.030773 | 0.511497 |
| rs11751591 | G | A | 0.0214 | 0.0034 | 2.70E-10 | SLE | 0.0725707 | 0.0431417 | 0.0925401 |
| rs11871043 | T | C | 0.0149 | 0.0025 | 1.70E-09 | SLE | -0.0618754 | 0.0327941 | 0.0591889 |
| rs12071912 | C | T | -0.015 | 0.0026 | 5.30E-09 | SLE | 0.0202027 | 0.0378225 | 0.593241 |
| rs12435797 | G | T | -0.017 | 0.0031 | 3.70E-08 | SLE | 0.040822 | 0.038954 | 0.29466 |
| rs12537376 | A | G | 0.0151 | 0.0025 | 1.70E-09 | SLE | -0.0099503 | 0.0375936 | 0.791254 |
| rs12765185 | T | A | -0.015 | 0.0027 | 3.90E-08 | SLE | 0.0725707 | 0.0314217 | 0.0209117 |
| rs13135092 | A | G | -0.033 | 0.0044 | 1.50E-13 | SLE | 0.105361 | 0.0563889 | 0.0616978 |
| rs13136239 | G | A | 0.0141 | 0.0026 | 3.60E-08 | SLE | -0.0099503 | 0.0247473 | 0.687627 |
| rs1443914 | T | C | 0.0162 | 0.0024 | 2.80E-11 | SLE | 0.0392207 | 0.0277853 | 0.158079 |
| rs17474406 | G | A | -0.049 | 0.0088 | 2.40E-08 | SLE | 0.040822 | 0.11518 | 0.723024 |
| rs1946247 | T | G | -0.019 | 0.0035 | 4.90E-08 | SLE | -0.0202027 | 0.0462323 | 0.662123 |
| rs197422 | C | A | -0.015 | 0.0025 | 2.00E-09 | SLE | -0.0295588 | 0.0296592 | 0.318952 |
| rs1976423 | A | C | -0.014 | 0.0024 | 8.20E-09 | SLE | -0.0295588 | 0.0295726 | 0.317537 |
| rs2006281 | C | T | 0.0135 | 0.0024 | 3.40E-08 | SLE | 0.0487902 | 0.0276912 | 0.07808 |
| rs2183271 | T | C | -0.014 | 0.0025 | 3.10E-08 | SLE | -0.0099503 | 0.0267229 | 0.709632 |
| rs2386584 | T | G | -0.017 | 0.0025 | 2.80E-11 | SLE | -0.0392207 | 0.0322094 | 0.223346 |
| rs2424248 | G | A | 0.023 | 0.0037 | 3.70E-10 | SLE | -0.0099503 | 0.0406354 | 0.806558 |
| rs28428925 | G | A | -0.021 | 0.0035 | 1.40E-09 | SLE | 0.0725707 | 0.0446623 | 0.104189 |
| rs285026 | G | T | -0.014 | 0.0025 | 1.90E-08 | SLE | 0.0198026 | 0.0346839 | 0.568036 |
| rs34811474 | G | A | 0.0192 | 0.0029 | 2.70E-11 | SLE | 0.105361 | 0.0413664 | 0.010865 |
| rs4852567 | A | G | 0.0149 | 0.0027 | 4.30E-08 | SLE | 0.0100503 | 0.0246136 | 0.683037 |
| rs59898460 | T | C | 0.0169 | 0.0025 | 9.20E-12 | SLE | 0.0100503 | 0.0344249 | 0.770325 |
| rs61883178 | C | A | -0.021 | 0.0033 | 2.00E-10 | SLE | -0.0392207 | 0.0347577 | 0.259149 |
| rs62098013 | G | A | -0.017 | 0.0026 | 4.00E-11 | SLE | -0.0392207 | 0.031131 | 0.20772 |
| rs6478241 | A | G | 0.0149 | 0.0025 | 3.10E-09 | SLE | -0.0202027 | 0.0279378 | 0.469599 |
| rs6869446 | T | C | -0.014 | 0.0025 | 9.50E-09 | SLE | -0.0676586 | 0.0278101 | 0.0149792 |
| rs6907508 | A | G | -0.022 | 0.0038 | 1.10E-08 | SLE | 0.0304592 | 0.0419708 | 0.468008 |
| rs6926377 | A | C | -0.016 | 0.0027 | 7.90E-09 | SLE | -0.0198026 | 0.0280805 | 0.48068 |
| rs6966540 | T | C | -0.014 | 0.0025 | 3.30E-08 | SLE | 0.0100503 | 0.023621 | 0.670483 |
| rs73581580 | G | A | -0.028 | 0.0037 | 5.30E-14 | SLE | 0.0512933 | 0.0503275 | 0.308113 |
| rs7628207 | T | C | 0.0195 | 0.0032 | 8.40E-10 | SLE | 0.0582689 | 0.0387082 | 0.132237 |
| rs7798894 | A | T | 0.0153 | 0.0027 | 1.60E-08 | SLE | -0.0304592 | 0.0283666 | 0.282926 |
| rs11871043 | T | C | 0.0149 | 0.0025 | 1.70E-09 | CeD | 0.0392207 | 0.0190519 | 0.0395303 |
| rs12435797 | G | T | -0.017 | 0.0031 | 3.70E-08 | CeD | 0.0432207 | 0.0230249 | 0.0605006 |
| rs6907508 | A | G | -0.022 | 0.0038 | 1.10E-08 | CeD | 0.0688143 | 0.0293972 | 0.0192402 |
| rs7628207 | T | C | 0.0195 | 0.0032 | 8.40E-10 | CeD | -0.0562529 | 0.0242177 | 0.0201902 |
| rs11871043 | T | C | 0.0149 | 0.0025 | 1.70E-09 | Psoriasis | -0.0070246 | 0.0348063 | 0.840057 |
| rs12435797 | G | T | -0.017 | 0.0031 | 3.70E-08 | Psoriasis | -0.0218593 | 0.0215981 | 0.311493 |
| rs6907508 | A | G | -0.022 | 0.0038 | 1.10E-08 | Psoriasis | 0.0732161 | 0.0756525 | 0.333147 |
| rs7628207 | T | C | 0.0195 | 0.0032 | 8.40E-10 | Psoriasis | 0.00319489 | 0.0637194 | 0.960011 |
| rs10259354 | G | A | 0.0147 | 0.0026 | 3.00E-08 | ALS | 0.0076 | 0.0149 | 0.6079 |
| rs10888692 | C | G | -0.014 | 0.0025 | 5.30E-09 | ALS | 0.0304 | 0.0138 | 0.0272402 |
| rs10992729 | C | T | 0.0158 | 0.0026 | 1.10E-09 | ALS | 0.0132 | 0.0144 | 0.3595 |
| rs11079993 | G | T | -0.017 | 0.0025 | 5.70E-12 | ALS | -0.0076 | 0.014 | 0.586001 |
| rs11599236 | T | C | 0.0138 | 0.0025 | 3.30E-08 | ALS | -0.0048 | 0.0138 | 0.730601 |
| rs11751591 | G | A | 0.0214 | 0.0034 | 2.70E-10 | ALS | -0.0042 | 0.0195 | 0.8306 |
| rs11786084 | G | A | -0.015 | 0.0026 | 2.30E-08 | ALS | -0.0109 | 0.0149 | 0.4633 |
| rs11871043 | T | C | 0.0149 | 0.0025 | 1.70E-09 | ALS | -0.0182 | 0.0138 | 0.1882 |
| rs12071912 | C | T | -0.015 | 0.0026 | 5.30E-09 | ALS | -0.0037 | 0.0164 | 0.8232 |
| rs12435797 | G | T | -0.017 | 0.0031 | 3.70E-08 | ALS | 0.0155 | 0.0167 | 0.3532 |
| rs12537376 | A | G | 0.0151 | 0.0025 | 1.70E-09 | ALS | -0.0077 | 0.014 | 0.581599 |
| rs12765185 | T | A | -0.015 | 0.0027 | 3.90E-08 | ALS | -0.0156 | 0.0155 | 0.3123 |
| rs13135092 | A | G | -0.033 | 0.0044 | 1.50E-13 | ALS | -0.0354 | 0.0252 | 0.1601 |
| rs13136239 | G | A | 0.0141 | 0.0026 | 3.60E-08 | ALS | 0.0192 | 0.0144 | 0.1818 |
| rs1443914 | T | C | 0.0162 | 0.0024 | 2.80E-11 | ALS | -0.0102 | 0.0136 | 0.4547 |
| rs17474406 | G | A | -0.049 | 0.0088 | 2.40E-08 | ALS | 0.0017 | 0.0527 | 0.9745 |
| rs1946247 | T | G | -0.019 | 0.0035 | 4.90E-08 | ALS | -0.0037 | 0.019 | 0.8457 |
| rs197422 | C | A | -0.015 | 0.0025 | 2.00E-09 | ALS | -0.0114 | 0.0139 | 0.4148 |
| rs1976423 | A | C | -0.014 | 0.0024 | 8.20E-09 | ALS | -0.0132 | 0.0177 | 0.4558 |
| rs2006281 | C | T | 0.0135 | 0.0024 | 3.40E-08 | ALS | 0.0075 | 0.0144 | 0.604999 |
| rs2183271 | T | C | -0.014 | 0.0025 | 3.10E-08 | ALS | -0.0073 | 0.0141 | 0.6042 |
| rs2386584 | T | G | -0.017 | 0.0025 | 2.80E-11 | ALS | -0.0243 | 0.0139 | 0.0803693 |
| rs2424248 | G | A | 0.023 | 0.0037 | 3.70E-10 | ALS | 0.017 | 0.0203 | 0.4042 |
| rs28428925 | G | A | -0.021 | 0.0035 | 1.40E-09 | ALS | -0.0011 | 0.0197 | 0.9537 |
| rs285026 | G | T | -0.014 | 0.0025 | 1.90E-08 | ALS | 0.0193 | 0.0136 | 0.1567 |
| rs34811474 | G | A | 0.0192 | 0.0029 | 2.70E-11 | ALS | -0.0355 | 0.0187 | 0.0582895 |
| rs4852567 | A | G | 0.0149 | 0.0027 | 4.30E-08 | ALS | 0.0197 | 0.015 | 0.1894 |
| rs59898460 | T | C | 0.0169 | 0.0025 | 9.20E-12 | ALS | -0.0301 | 0.0139 | 0.0303201 |
| rs61883178 | C | A | -0.021 | 0.0033 | 2.00E-10 | ALS | -0.0109 | 0.0177 | 0.5372 |
| rs62098013 | G | A | -0.017 | 0.0026 | 4.00E-11 | ALS | -0.0055 | 0.0146 | 0.706401 |
| rs6478241 | A | G | 0.0149 | 0.0025 | 3.10E-09 | ALS | -2.00E-04 | 0.0141 | 0.9886 |
| rs6770476 | C | T | -0.015 | 0.0027 | 9.40E-09 | ALS | -0.0017 | 0.0149 | 0.9105 |
| rs6869446 | T | C | -0.014 | 0.0025 | 9.50E-09 | ALS | 2.00E-04 | 0.0141 | 0.9873 |
| rs6907508 | A | G | -0.022 | 0.0038 | 1.10E-08 | ALS | -0.0438 | 0.0213 | 0.0397 |
| rs6926377 | A | C | -0.016 | 0.0027 | 7.90E-09 | ALS | -0.0352 | 0.0146 | 0.0160702 |
| rs6966540 | T | C | -0.014 | 0.0025 | 3.30E-08 | ALS | -0.0048 | 0.014 | 0.729899 |
| rs73581580 | G | A | -0.028 | 0.0037 | 5.30E-14 | ALS | -0.005 | 0.0267 | 0.8529 |
| rs7628207 | T | C | 0.0195 | 0.0032 | 8.40E-10 | ALS | -0.0253 | 0.0178 | 0.1547 |
| rs7798894 | A | T | 0.0153 | 0.0027 | 1.60E-08 | ALS | 0.0152 | 0.015 | 0.3105 |
| rs10259354 | G | A | 0.0147 | 0.0026 | 3.00E-08 | T1D | -0.0089 | 0.0256 | 0.7287 |
| rs10888692 | C | G | -0.014 | 0.0025 | 5.30E-09 | T1D | 0.0132 | 0.0234 | 0.5733 |
| rs10992729 | C | T | 0.0158 | 0.0026 | 1.10E-09 | T1D | -0.0089 | 0.0247 | 0.7196 |
| rs11079993 | G | T | -0.017 | 0.0025 | 5.70E-12 | T1D | -0.0201 | 0.0239 | 0.3992 |
| rs11599236 | T | C | 0.0138 | 0.0025 | 3.30E-08 | T1D | 0.0028 | 0.0239 | 0.9059 |
| rs11751591 | G | A | 0.0214 | 0.0034 | 2.70E-10 | T1D | 0.0816 | 0.0339 | 0.0161901 |
| rs11786084 | G | A | -0.015 | 0.0026 | 2.30E-08 | T1D | 0.0031 | 0.0263 | 0.9065 |
| rs11871043 | T | C | 0.0149 | 0.0025 | 1.70E-09 | T1D | -0.0012 | 0.0236 | 0.9597 |
| rs12071912 | C | T | -0.015 | 0.0026 | 5.30E-09 | T1D | 0.0094 | 0.026 | 0.7173 |
| rs12435797 | G | T | -0.017 | 0.0031 | 3.70E-08 | T1D | 0.0337 | 0.0299 | 0.259 |
| rs12537376 | A | G | 0.0151 | 0.0025 | 1.70E-09 | T1D | 0.011 | 0.0244 | 0.6524 |
| rs12765185 | T | A | -0.015 | 0.0027 | 3.90E-08 | T1D | 0.0529 | 0.0269 | 0.0493696 |
| rs13135092 | A | G | -0.033 | 0.0044 | 1.50E-13 | T1D | 0.0349 | 0.0426 | 0.4126 |
| rs13136239 | G | A | 0.0141 | 0.0026 | 3.60E-08 | T1D | 0.0148 | 0.0248 | 0.551001 |
| rs1443914 | T | C | 0.0162 | 0.0024 | 2.80E-11 | T1D | -0.0211 | 0.0235 | 0.3685 |
| rs17474406 | G | A | -0.049 | 0.0088 | 2.40E-08 | T1D | 0.061 | 0.0841 | 0.4682 |
| rs1946247 | T | G | -0.019 | 0.0035 | 4.90E-08 | T1D | 0.0297 | 0.0329 | 0.3663 |
| rs197422 | C | A | -0.015 | 0.0025 | 2.00E-09 | T1D | 0.0057 | 0.0242 | 0.8149 |
| rs1976423 | A | C | -0.014 | 0.0024 | 8.20E-09 | T1D | 0.0051 | 0.0237 | 0.8303 |
| rs2006281 | C | T | 0.0135 | 0.0024 | 3.40E-08 | T1D | 0.0188 | 0.0243 | 0.4385 |
| rs2183271 | T | C | -0.014 | 0.0025 | 3.10E-08 | T1D | -0.0432 | 0.0244 | 0.0766002 |
| rs2386584 | T | G | -0.017 | 0.0025 | 2.80E-11 | T1D | 0.007 | 0.0239 | 0.7676 |
| rs2424248 | G | A | 0.023 | 0.0037 | 3.70E-10 | T1D | -0.0209 | 0.0351 | 0.5516 |
| rs28428925 | G | A | -0.021 | 0.0035 | 1.40E-09 | T1D | -0.0084 | 0.0334 | 0.8008 |
| rs285026 | G | T | -0.014 | 0.0025 | 1.90E-08 | T1D | -0.0255 | 0.0235 | 0.2783 |
| rs34811474 | G | A | 0.0192 | 0.0029 | 2.70E-11 | T1D | 0.0368 | 0.0326 | 0.2593 |
| rs4852567 | A | G | 0.0149 | 0.0027 | 4.30E-08 | T1D | 0.0404 | 0.0263 | 0.1243 |
| rs59898460 | T | C | 0.0169 | 0.0025 | 9.20E-12 | T1D | 0.0275 | 0.024 | 0.2511 |
| rs61883178 | C | A | -0.021 | 0.0033 | 2.00E-10 | T1D | -7.00E-04 | 0.0306 | 0.9828 |
| rs62098013 | G | A | -0.017 | 0.0026 | 4.00E-11 | T1D | 0.0093 | 0.0245 | 0.703399 |
| rs6478241 | A | G | 0.0149 | 0.0025 | 3.10E-09 | T1D | 0.0041 | 0.0241 | 0.8646 |
| rs6770476 | C | T | -0.015 | 0.0027 | 9.40E-09 | T1D | 0.0955 | 0.0258 | 0.0002138 |
| rs6869446 | T | C | -0.014 | 0.0025 | 9.50E-09 | T1D | 0.0024 | 0.0243 | 0.9202 |
| rs6907508 | A | G | -0.022 | 0.0038 | 1.10E-08 | T1D | -0.027 | 0.0358 | 0.4516 |
| rs6926377 | A | C | -0.016 | 0.0027 | 7.90E-09 | T1D | -0.0095 | 0.026 | 0.715801 |
| rs6966540 | T | C | -0.014 | 0.0025 | 3.30E-08 | T1D | -0.0428 | 0.0245 | 0.0810103 |
| rs73581580 | G | A | -0.028 | 0.0037 | 5.30E-14 | T1D | -0.0463 | 0.0453 | 0.3065 |
| rs7628207 | T | C | 0.0195 | 0.0032 | 8.40E-10 | T1D | -0.0032 | 0.0303 | 0.9149 |
| rs7798894 | A | T | 0.0153 | 0.0027 | 1.60E-08 | T1D | 0.0166 | 0.0257 | 0.5179 |
| rs11871043 | T | C | 0.0149 | 0.0025 | 1.70E-09 | IBD | 0.0173806 | 0.0103493 | 0.0930743 |
| rs12435797 | G | T | -0.017 | 0.0031 | 3.70E-08 | IBD | 0.00949856 | 0.0128438 | 0.459577 |
| rs6907508 | A | G | -0.022 | 0.0038 | 1.10E-08 | IBD | 0.0199632 | 0.0160931 | 0.214796 |
| rs7628207 | T | C | 0.0195 | 0.0032 | 8.40E-10 | IBD | -0.0518811 | 0.0130134 | 6.70E-05 |
| rs10259354 | G | A | 0.0147 | 0.0026 | 3.00E-08 | RA | 0.0100503 | 0.025198 | 0.69 |
| rs10888692 | C | G | -0.014 | 0.0025 | 5.30E-09 | RA | -0.0099503 | 0.0356235 | 0.780001 |
| rs10992729 | C | T | 0.0158 | 0.0026 | 1.10E-09 | RA | 0.0100503 | 0.0343748 | 0.77 |
| rs11079993 | G | T | -0.017 | 0.0025 | 5.70E-12 | RA | -0.0198026 | 0.0183303 | 0.28 |
| rs11599236 | T | C | 0.0138 | 0.0025 | 3.30E-08 | RA | 0.0198026 | 0.019913 | 0.32 |
| rs11751591 | G | A | 0.0214 | 0.0034 | 2.70E-10 | RA | -0.0099503 | 0.0151029 | 0.51 |
| rs11786084 | G | A | -0.015 | 0.0026 | 2.30E-08 | RA | -0.0198026 | 0.0175806 | 0.26 |
| rs12435797 | G | T | -0.017 | 0.0031 | 3.70E-08 | RA | 0.0202027 | 0.023502 | 0.39 |
| rs12537376 | A | G | 0.0151 | 0.0025 | 1.70E-09 | RA | 0.0392207 | 0.0199232 | 0.0490004 |
| rs12765185 | T | A | -0.015 | 0.0027 | 3.90E-08 | RA | -0.0099503 | 0.0200672 | 0.62 |
| rs13135092 | A | G | -0.033 | 0.0044 | 1.50E-13 | RA | 0.0295588 | 0.040913 | 0.47 |
| rs13136239 | G | A | 0.0141 | 0.0026 | 3.60E-08 | RA | -0.0099503 | 0.0249472 | 0.69 |
| rs1443914 | T | C | 0.0162 | 0.0024 | 2.80E-11 | RA | -0.0304592 | 0.0206392 | 0.14 |
| rs17474406 | G | A | -0.049 | 0.0088 | 2.40E-08 | RA | 0.0202027 | 0.063403 | 0.75 |
| rs1946247 | T | G | -0.019 | 0.0035 | 4.90E-08 | RA | 0.00995033 | 0.0392755 | 0.8 |
| rs197422 | C | A | -0.015 | 0.0025 | 2.00E-09 | RA | -0.0198026 | 0.0262142 | 0.450001 |
| rs2006281 | C | T | 0.0135 | 0.0024 | 3.40E-08 | RA | -0.0295588 | 0.0285197 | 0.3 |
| rs2183271 | T | C | -0.014 | 0.0025 | 3.10E-08 | RA | 0.0198026 | 0.0191065 | 0.3 |
| rs2386584 | T | G | -0.017 | 0.0025 | 2.80E-11 | RA | -0.0202027 | 0.0164715 | 0.22 |
| rs2424248 | G | A | 0.023 | 0.0037 | 3.70E-10 | RA | 0.0304592 | 0.0281946 | 0.28 |
| rs28428925 | G | A | -0.021 | 0.0035 | 1.40E-09 | RA | -0.0099503 | 0.0312276 | 0.75 |
| rs285026 | G | T | -0.014 | 0.0025 | 1.90E-08 | RA | -0.0198026 | 0.0235291 | 0.4 |
| rs4852567 | A | G | 0.0149 | 0.0027 | 4.30E-08 | RA | -0.0100503 | 0.0181616 | 0.58 |
| rs59898460 | T | C | 0.0169 | 0.0025 | 9.20E-12 | RA | 0.0198026 | 0.0235291 | 0.4 |
| rs61883178 | C | A | -0.021 | 0.0033 | 2.00E-10 | RA | -0.0392207 | 0.0245407 | 0.11 |
| rs62098013 | G | A | -0.017 | 0.0026 | 4.00E-11 | RA | -0.0099503 | 0.0162371 | 0.54 |
| rs6478241 | A | G | 0.0149 | 0.0025 | 3.10E-09 | RA | 0.0198026 | 0.0191065 | 0.3 |
| rs6770476 | C | T | -0.015 | 0.0027 | 9.40E-09 | RA | -0.0099503 | 0.0151029 | 0.51 |
| rs6869446 | T | C | -0.014 | 0.0025 | 9.50E-09 | RA | -0.040822 | 0.0217047 | 0.0599998 |
| rs6907508 | A | G | -0.022 | 0.0038 | 1.10E-08 | RA | -0.0725707 | 0.0235948 | 0.0021 |
| rs6966540 | T | C | -0.014 | 0.0025 | 3.30E-08 | RA | 0.0198026 | 0.0220895 | 0.37 |
| rs7628207 | T | C | 0.0195 | 0.0032 | 8.40E-10 | RA | 0.00995033 | 0.0356235 | 0.780001 |
| rs7798894 | A | T | 0.0153 | 0.0027 | 1.60E-08 | RA | -0.0202027 | 0.0187007 | 0.28 |
| rs10259354 | G | A | 0.0147 | 0.0026 | 3.00E-08 | MS | 0.00843548 | 0.0179609 | 0.6386 |
| rs10888692 | C | G | -0.014 | 0.0025 | 5.30E-09 | MS | -0.0008003 | 0.0166228 | 0.9616 |
| rs10992729 | C | T | 0.0158 | 0.0026 | 1.10E-09 | MS | 0.0166376 | 0.0173181 | 0.3367 |
| rs11079993 | G | T | -0.017 | 0.0025 | 5.70E-12 | MS | -0.0113355 | 0.0166968 | 0.4972 |
| rs11599236 | T | C | 0.0138 | 0.0025 | 3.30E-08 | MS | -0.0003999 | 0.0192209 | 0.9834 |
| rs11751591 | G | A | 0.0214 | 0.0034 | 2.70E-10 | MS | -0.0147076 | 0.0233401 | 0.5286 |
| rs11786084 | G | A | -0.015 | 0.0026 | 2.30E-08 | MS | 0.0116678 | 0.0175517 | 0.5062 |
| rs11871043 | T | C | 0.0149 | 0.0025 | 1.70E-09 | MS | -0.0050125 | 0.0165438 | 0.7619 |
| rs12071912 | C | T | -0.015 | 0.0026 | 5.30E-09 | MS | -0.0422814 | 0.028715 | 0.1409 |
| rs12435797 | G | T | -0.017 | 0.0031 | 3.70E-08 | MS | -0.027474 | 0.0203128 | 0.1762 |
| rs12765185 | T | A | -0.015 | 0.0027 | 3.90E-08 | MS | -0.0209784 | 0.01907 | 0.2713 |
| rs13135092 | A | G | -0.033 | 0.0044 | 1.50E-13 | MS | -0.113505 | 0.037458 | 0.00244399 |
| rs13136239 | G | A | 0.0141 | 0.0026 | 3.60E-08 | MS | 0.047742 | 0.0172903 | 0.00575904 |
| rs1443914 | T | C | 0.0162 | 0.0024 | 2.80E-11 | MS | 0.00259663 | 0.016053 | 0.8715 |
| rs17474406 | G | A | -0.049 | 0.0088 | 2.40E-08 | MS | 0.0249853 | 0.0685505 | 0.715501 |
| rs1946247 | T | G | -0.019 | 0.0035 | 4.90E-08 | MS | -0.0246926 | 0.0228378 | 0.2796 |
| rs197422 | C | A | -0.015 | 0.0025 | 2.00E-09 | MS | 0.0247026 | 0.0166625 | 0.1382 |
| rs1976423 | A | C | -0.014 | 0.0024 | 8.20E-09 | MS | -0.0205866 | 0.0172573 | 0.2329 |
| rs2006281 | C | T | 0.0135 | 0.0024 | 3.40E-08 | MS | -0.0073268 | 0.0173775 | 0.673301 |
| rs2386584 | T | G | -0.017 | 0.0025 | 2.80E-11 | MS | -0.0159262 | 0.016605 | 0.3375 |
| rs2424248 | G | A | 0.023 | 0.0037 | 3.70E-10 | MS | -0.0027961 | 0.0241956 | 0.908 |
| rs28428925 | G | A | -0.021 | 0.0035 | 1.40E-09 | MS | 0.00836492 | 0.0267047 | 0.7541 |
| rs285026 | G | T | -0.014 | 0.0025 | 1.90E-08 | MS | -0.0191828 | 0.0163468 | 0.2406 |
| rs34811474 | G | A | 0.0192 | 0.0029 | 2.70E-11 | MS | 0.0803811 | 0.0422883 | 0.0573297 |
| rs4852567 | A | G | 0.0149 | 0.0027 | 4.30E-08 | MS | 0.031111 | 0.0181552 | 0.0866004 |
| rs59898460 | T | C | 0.0169 | 0.0025 | 9.20E-12 | MS | 0.00019998 | 0.018995 | 0.9916 |
| rs61883178 | C | A | -0.021 | 0.0033 | 2.00E-10 | MS | 0.0251803 | 0.0215627 | 0.2429 |
| rs6478241 | A | G | 0.0149 | 0.0025 | 3.10E-09 | MS | 0.0030952 | 0.0169022 | 0.8547 |
| rs6770476 | C | T | -0.015 | 0.0027 | 9.40E-09 | MS | 0.0265446 | 0.0180094 | 0.1405 |
| rs6869446 | T | C | -0.014 | 0.0025 | 9.50E-09 | MS | -0.0185714 | 0.0170233 | 0.2753 |
| rs6907508 | A | G | -0.022 | 0.0038 | 1.10E-08 | MS | -0.0058169 | 0.024738 | 0.8141 |
| rs6926377 | A | C | -0.016 | 0.0027 | 7.90E-09 | MS | -0.0087381 | 0.0177084 | 0.6217 |
| rs6966540 | T | C | -0.014 | 0.0025 | 3.30E-08 | MS | -0.0085632 | 0.0170068 | 0.614601 |
| rs7628207 | T | C | 0.0195 | 0.0032 | 8.40E-10 | MS | -0.0082657 | 0.0213394 | 0.698499 |
| rs7798894 | A | T | 0.0153 | 0.0027 | 1.60E-08 | MS | 0.00863719 | 0.0181414 | 0.634 |

Abbreviations: MCP, Multisite Chronic Pain; Effect allele, EA; other allele, OA; AIDs, Autoimmune Diseases; beta, estimate coefficient; se, standard error of coefficient estimate; ALS, amyotrophic lateral sclerosis; CeD, celiac disease; IBD, inflammatory bowel disease; MS, multiple sclerosis; RA, rheumatoid arthritis; SLE, systemic lupus erythematosus; T1D, Type 1 diabetes.

## Supplementary Table S2 Harmonies data for CWP and AIDs.

| SNP | EA | OA | Exposure (CWP) | | | Outcome (AIDs) | | | |
| --- | --- | --- | --- | --- | --- | --- | --- | --- | --- |
|  |  |  | beta | se | *P*-value | outcome | beta | se | *P*-value |
| rs10490825 | G | A | -0.0039 | 7.00E-04 | 1.30E-08 | SLE | 0.0304592 | 0.0456656 | 0.504769 |
| rs1491985 | G | C | 0.0034 | 6.00E-04 | 1.60E-08 | SLE | 0.0582689 | 0.0354088 | 0.0998458 |
| rs165599 | G | A | -0.0028 | 5.00E-04 | 2.50E-08 | SLE | 0.0295588 | 0.0273612 | 0.28 |
| rs1491985 | G | C | 0.0034 | 6.00E-04 | 1.60E-08 | CeD | -0.0562529 | 0.0241827 | 0.0200101 |
| rs1491985 | G | C | 0.0034 | 6.00E-04 | 1.60E-08 | Psoriasis | 0.00796817 | 0.0453993 | 0.860676 |
| rs10490825 | G | A | -0.0039 | 7.00E-04 | 1.30E-08 | ALS | -0.0117 | 0.0206 | 0.5703 |
| rs1491985 | G | C | 0.0034 | 6.00E-04 | 1.60E-08 | ALS | -0.0265 | 0.0178 | 0.1352 |
| rs1491985 | G | C | 0.0034 | 6.00E-04 | 1.60E-08 | ALS | -0.0265 | 0.0178 | 0.1352 |
| rs165599 | G | A | -0.0028 | 5.00E-04 | 2.50E-08 | ALS | 0.0263 | 0.0148 | 0.0751104 |
| rs10490825 | G | A | -0.0039 | 7.00E-04 | 1.30E-08 | T1D | -0.0191 | 0.0343 | 0.577301 |
| rs1491985 | G | C | 0.0034 | 6.00E-04 | 1.60E-08 | T1D | -0.0038 | 0.0303 | 0.8995 |
| rs165599 | G | A | -0.0028 | 5.00E-04 | 2.50E-08 | T1D | 0.0272 | 0.0255 | 0.2858 |
| rs1491985 | G | C | 0.0034 | 6.00E-04 | 1.60E-08 | IBD | -0.0477016 | 0.0130035 | 0.00024409 |
| rs10490825 | G | A | -0.0039 | 7.00E-04 | 1.30E-08 | RA | -0.0099503 | 0.0437291 | 0.82 |
| rs1491985 | G | C | 0.0034 | 6.00E-04 | 1.60E-08 | RA | 0.0100503 | 0.0377392 | 0.79 |
| rs165599 | G | A | -0.0028 | 5.00E-04 | 2.50E-08 | RA | 0.0100503 | 0.0208631 | 0.630001 |
| rs10490825 | G | A | -0.0039 | 7.00E-04 | 1.30E-08 | MS | -0.0218367 | 0.0232731 | 0.3481 |
| rs1491985 | G | C | 0.0034 | 6.00E-04 | 1.60E-08 | MS | -0.0085632 | 0.0213895 | 0.6889 |
| rs165599 | G | A | -0.0028 | 5.00E-04 | 2.50E-08 | MS | -0.0016986 | 0.0180675 | 0.9251 |
| rs10490825 | G | A | -0.0039 | 7.00E-04 | 1.30E-08 | SLE | 0.0304592 | 0.0456656 | 0.504769 |
| rs1491985 | G | C | 0.0034 | 6.00E-04 | 1.60E-08 | SLE | 0.0582689 | 0.0354088 | 0.0998458 |
| rs165599 | G | A | -0.0028 | 5.00E-04 | 2.50E-08 | SLE | 0.0295588 | 0.0273612 | 0.28 |

Abbreviations: CWP, chronic widespread pain; Effect allele, EA; other allele, OA; AIDs, Autoimmune Diseases; beta, estimate coefficient; se, standard error of coefficient estimate; ALS, amyotrophic lateral sclerosis; CeD, celiac disease; IBD, inflammatory bowel disease; MS, multiple sclerosis; RA, rheumatoid arthritis; SLE, systemic lupus erythematosus; T1D, Type 1 diabetes.

## Supplementary Table S3 MR results for MCP and AIDs.

| **Outcome** | **Method** | **N snp** | **b** | **se** | **OR (95%CI)** | ***P*-value** |
| --- | --- | --- | --- | --- | --- | --- |
| ALS | IVW | 38 | 0.22797789 | 0.15851966 | 1.26 (0.92, 1.71) | 0.150 |
| ALS | MR Egger | 38 | 0.34134572 | 0.78636892 | 1.41 (0.3, 6.57) | 0.667 |
| ALS | Simple mode | 38 | 0.40845986 | 0.50734738 | 1.5 (0.56, 4.07) | 0.426 |
| ALS | Weighted median | 38 | 0.33291195 | 0.21232363 | 1.4 (0.92, 2.12) | 0.117 |
| ALS | Weighted mode | 38 | 0.40845986 | 0.46268889 | 1.5 (0.61, 3.73) | 0.383 |
| CeD | MR Egger | 4 | -16.929701 | 5.25498926 | NA | 0.084 |
| CeD | IVW | 4 | -1.4343835 | 1.39140224 | 0.24 (0.02, 3.64) | 0.303 |
| CeD | Simple mode | 4 | -2.8869095 | 0.95851589 | 0.06 (0.01, 0.36) | 0.057 |
| CeD | Weighted median | 4 | -2.6733581 | 0.82482579 | 0.07 (0.01, 0.35) | 0.001 |
| CeD | Weighted mode | 4 | -2.8715578 | 0.96555974 | 0.06 (0.01, 0.38) | 0.059 |
| IBD | IVW | 4 | -0.7829476 | 0.81714398 | 0.46 (0.09, 2.27) | 0.338 |
| IBD | MR Egger | 4 | -8.517093 | 4.5748393 | NA | 0.204 |
| IBD | Simple mode | 4 | -0.7172287 | 0.90026087 | 0.49 (0.08, 2.85) | 0.484 |
| IBD | Weighted median | 4 | -0.6953738 | 0.51385869 | 0.50 (0.18, 1.37) | 0.176 |
| IBD | Weighted mode | 4 | -0.6576 | 0.88142892 | 0.52 (0.09, 2.92) | 0.510 |
| MS | MR Egger | 32 | 0.31620789 | 1.17929294 | 1.37 (0.14, 13.84) | 0.790 |
| MS | IVW | 32 | 0.46200349 | 0.22918099 | 1.59 (1.01, 2.49) | 0.044 |
| MS | Simple mode | 32 | 0.07831165 | 0.59920305 | 1.08 (0.33, 3.50) | 0.897 |
| MS | Weighted median | 32 | 0.24083638 | 0.29745409 | 1.27 (0.71, 2.28) | 0.418 |
| MS | Weighted mode | 32 | 0.17056033 | 0.57236972 | 1.19 (0.39, 3.64) | 0.768 |
| Psoriasis | MR Egger | 4 | -3.794241 | 10.4276024 | NA | 0.751 |
| Psoriasis | IVW | 4 | 0.4616712 | 0.99960999 | 1.59 (0.22, 11.26) | 0.644 |
| Psoriasis | Simple mode | 4 | 0.17512977 | 1.82854303 | 1.19 (0.03, 42.91) | 0.930 |
| Psoriasis | Weighted median | 4 | 0.71482153 | 1.15002863 | 2.04 (0.21, 19.47) | 0.534 |
| Psoriasis | Weighted mode | 4 | 1.08579363 | 1.24533932 | 2.96 (0.26, 34.01) | 0.447 |
| RA | MR Egger | 30 | 0.11426465 | 1.16332871 | 1.12 (0.11, 10.96) | 0.922 |
| RA | IVW | 30 | 0.53953582 | 0.24500533 | 1.72 (1.06, 2.77) | 0.028 |
| RA | Simple mode | 30 | 1.05839914 | 0.7292144 | 2.88 (0.69, 12.03) | 0.157 |
| RA | Weighted median | 30 | 0.63986468 | 0.34413082 | 1.90 (0.97, 3.72) | 0.063 |
| RA | Weighted mode | 30 | 1.04024693 | 0.6516611 | 2.83 (0.79, 10.15) | 0.121 |
| SLE | MR Egger | 34 | -2.3983947 | 1.82090713 | 0.09 (0, 3.22) | 0.197 |
| SLE | IVW | 34 | 0.57886735 | 0.39635313 | 1.78 (0.82, 3.88) | 0.144 |
| SLE | Simple mode | 34 | 1.83663878 | 1.29092508 | 6.28 (0.5, 78.8) | 0.164 |
| SLE | Weighted median | 34 | 0.68171062 | 0.51474366 | 1.98 (0.72, 5.42) | 0.185 |
| SLE | Weighted mode | 34 | 1.77428047 | 1.09359073 | 5.9 (0.69, 50.29) | 0.114 |
| T1D | MR Egger | 38 | -0.5600407 | 1.39704495 | 0.57 (0.04, 8.83) | 0.691 |
| T1D | IVW | 38 | 0.14015184 | 0.28810658 | 1.15 (0.65, 2.02) | 0.627 |
| T1D | Simple mode | 38 | -0.2420166 | 0.75649429 | 0.79 (0.18, 3.46) | 0.751 |
| T1D | Weighted median | 38 | -0.0676113 | 0.36839753 | 0.93 (0.45, 1.92) | 0.854 |
| T1D | Weighted mode | 38 | -0.2972103 | 0.71071073 | 0.74 (0.18, 2.99) | 0.678 |

Abbreviations: MCP, multisite chronic pain; ALS, amyotrophic lateral sclerosis; CeD, celiac disease; IBD, inflammatory bowel disease; MS, multiple sclerosis; RA, rheumatoid arthritis; SLE, systemic lupus erythematosus; T1D, Type 1 diabetes; IVW, Inverse variance weighted; beta, estimate coefficient; se, standard error of coefficient estimate; OR, odds ratio; CI, confidence interval.

## Supplementary Table S4 Heterogeneity for MCP and AIDs.

| **Exposure** | **Outcome** | **Method** | **Q** | **Q_df** | ***P*-value** |
| --- | --- | --- | --- | --- | --- |
| MCP | ALS | MR Egger | 39.5192514 | 36 | 0.31561125 |
| MCP | ALS | IVW | 39.5430609 | 37 | 0.35707968 |
| MCP | CeD | MR Egger | 2.53287019 | 2 | 0.28183455 |
| MCP | CeD | IVW | 13.7615461 | 3 | 0.00324837 |
| MCP | IBD | MR Egger | 6.48097159 | 2 | 0.03914487 |
| MCP | IBD | IVW | 15.9264517 | 3 | 0.00117404 |
| MCP | MS | MR Egger | 37.8959266 | 30 | 0.15244 |
| MCP | MS | IVW | 37.9160173 | 31 | 0.18307247 |
| MCP | Psoriasis | MR Egger | 1.62277679 | 2 | 0.44424086 |
| MCP | Psoriasis | IVW | 1.79089927 | 3 | 0.61691736 |
| MCP | RA | MR Egger | 31.7754009 | 28 | 0.28374683 |
| MCP | RA | IVW | 31.9343214 | 29 | 0.32276363 |
| MCP | SLE | MR Egger | 42.0656796 | 32 | 0.10978339 |
| MCP | SLE | IVW | 45.7452844 | 33 | 0.0690753 |
| MCP | T1D | MR Egger | 44.3932714 | 36 | 0.15900668 |
| MCP | T1D | IVW | 44.7170855 | 37 | 0.17938847 |

Abbreviations: MCP, multisite chronic pain; ALS, amyotrophic lateral sclerosis; CeD, celiac disease; IBD, inflammatory bowel disease; MS, multiple sclerosis; RA, rheumatoid arthritis; SLE, systemic lupus erythematosus; T1D, Type 1 diabetes; IVW, Inverse variance weighted

## Supplementary Table S5 Pleiotropy for MCP and AIDs.

| **Exposure** | **Outcome** | **egger_intercept** | **se** | ***P*-value** |
| --- | --- | --- | --- | --- |
| MCP | ALS | -0.001929 | 0.01309801 | 0.88373861 |
| MCP | CeD | 0.27805704 | 0.09338155 | 0.09670281 |
| MCP | IBD | 0.13883182 | 0.08131713 | 0.22989046 |
| MCP | MS | 0.00246245 | 0.01952565 | 0.90048336 |
| MCP | Psoriasis | 0.07346074 | 0.17916055 | 0.72153476 |
| MCP | RA | 0.00744711 | 0.0199005 | 0.71106194 |
| MCP | SLE | 0.05089441 | 0.03041995 | 0.10406554 |
| MCP | T1D | 0.01193225 | 0.02328529 | 0.61147502 |

Abbreviations: se, standard error of coefficient estimate; MCP, multisite chronic pain; ALS, amyotrophic lateral sclerosis; CeD, celiac disease; IBD, inflammatory bowel disease; MS, multiple sclerosis; RA, rheumatoid arthritis; SLE, systemic lupus erythematosus; T1D, Type 1 diabetes.

## Supplementary Table S6 MR-PRESSO global test for Multisite chronic pain and AIDs.

| **Exposure** | **Outcome** | **MR-PRESSO global test** | | **MR-PRESSO Outlier test** | **MR-PRESSO distortion test** | |
| --- | --- | --- | --- | --- | --- | --- |
|  |  | **RSSobs** | ***P*-value** |  | **Distortion Coefficient** | ***P*-value** |
| MCP | ALS | 47.8899 | 0.188 | None | None | None |
|  | CeD | 24.8373 | 0.028 | rs11871043 | 49.6606 | 0.582 |
|  | IBD | 30.0092 | 0.02 | rs11871043, rs7628207 | -6.5661 | <0.001 |
|  | MS | 40.7744 | 0.282 | None | None | None |
|  | RA | 36.7682 | 0.35 | None | None | None |
|  | SLE | 58.2099 | 0.026 | No significant outliers | None | None |
|  | T1D | 47.4168 | 0.196 | None | None | None |
|  | Psoriasis | 4.88895 | 0.596 | None | None | None |

Abbreviations: MR-PRESSO test, MR Pleiotropy Residual Sum and Outlier test; MCP, multisite chronic pain; ALS, amyotrophic lateral sclerosis; CeD, celiac disease; IBD, inflammatory bowel disease; MS, multiple sclerosis; RA, rheumatoid arthritis; SLE, systemic lupus erythematosus; T1D, Type 1 diabetes.

## Supplementary Table S7 Harmonies data for MCP and BMI.

| **SNP** | **EA** | **OA** | **Exposure (MCP)** | | | **Outcome (BMI)** | | |
| --- | --- | --- | --- | --- | --- | --- | --- | --- |
|  |  |  | **beta** | **se** | ***P*-value** | **beta** | **se** | ***P*-value** |
| rs10259354 | G | A | 0.0147 | 0.003 | 3.00E-08 | -0.0095 | 0.0055 | 0.0841201 |
| rs10888692 | C | G | -0.0143 | 0.003 | 5.30E-09 | 0.0011 | 0.0051 | 0.8292 |
| rs10992729 | C | T | 0.0158 | 0.003 | 1.10E-09 | -0.0025 | 0.0052 | 0.6307 |
| rs11079993 | G | T | -0.0173 | 0.003 | 5.70E-12 | 8.00E-04 | 0.0052 | 0.8777 |
| rs11599236 | T | C | 0.0138 | 0.003 | 3.30E-08 | 0.0106 | 0.0051 | 0.0376704 |
| rs11751591 | G | A | 0.0214 | 0.003 | 2.70E-10 | 0.0181 | 0.0074 | 0.0144501 |
| rs11871043 | T | C | 0.0149 | 0.003 | 1.70E-09 | -0.0044 | 0.0041 | 0.2808 |
| rs12435797 | G | T | -0.0173 | 0.003 | 3.70E-08 | -0.0068 | 0.0063 | 0.2804 |
| rs13136239 | G | A | 0.0141 | 0.003 | 3.60E-08 | 0.0097 | 0.0052 | 0.0621298 |
| rs1443914 | T | C | 0.0162 | 0.002 | 2.80E-11 | 0.0028 | 0.005 | 0.5755 |
| rs1946247 | T | G | -0.019 | 0.004 | 4.90E-08 | 0.0017 | 0.0071 | 0.8108 |
| rs197422 | C | A | -0.015 | 0.003 | 2.00E-09 | -0.0103 | 0.0051 | 0.04342 |
| rs1976423 | A | C | -0.014 | 0.002 | 8.20E-09 | -0.0049 | 0.0054 | 0.3642 |
| rs2183271 | T | C | -0.014 | 0.003 | 3.10E-08 | -0.0091 | 0.0062 | 0.1422 |
| rs2386584 | T | G | -0.0166 | 0.003 | 2.80E-11 | -0.0015 | 0.0042 | 0.7106 |
| rs2424248 | G | A | 0.023 | 0.004 | 3.70E-10 | -0.0036 | 0.0076 | 0.6357 |
| rs285026 | G | T | -0.0138 | 0.003 | 1.90E-08 | 0.0016 | 0.005 | 0.749 |
| rs4852567 | A | G | 0.0149 | 0.003 | 4.30E-08 | -0.0039 | 0.0057 | 0.4938 |
| rs61883178 | C | A | -0.0208 | 0.003 | 2.00E-10 | -0.0017 | 0.0068 | 0.8026 |
| rs6478241 | A | G | 0.0149 | 0.003 | 3.10E-09 | -0.0017 | 0.0052 | 0.7437 |
| rs6869446 | T | C | -0.0144 | 0.003 | 9.50E-09 | -9.00E-04 | 0.0053 | 0.8652 |
| rs6907508 | A | G | -0.0217 | 0.004 | 1.10E-08 | -0.011 | 0.007 | 0.1161 |
| rs6926377 | A | C | -0.0155 | 0.003 | 7.90E-09 | -9.00E-04 | 0.0055 | 0.87 |
| rs6966540 | T | C | -0.0139 | 0.003 | 3.30E-08 | -0.0071 | 0.0054 | 0.1886 |
| rs7628207 | T | C | 0.0195 | 0.003 | 8.40E-10 | 0.016 | 0.0067 | 0.0169399 |
| rs7798894 | A | T | 0.0153 | 0.003 | 1.60E-08 | 0.0016 | 0.0057 | 0.778901 |

Abbreviations: MCP, Multisite Chronic Pain; BMI, Body Mass Index; EA, Effect allele; OA, other allele; beta, estimate coefficient; se, standard error of coefficient estimate.

## Supplementary Table S8 Harmonies data for BMI and RA/MS.

| **SNP** | **EA** | **OA** | **Exposure (BMI)** | | | **Outcome (RA/MS)** | | | |
| --- | --- | --- | --- | --- | --- | --- | --- | --- | --- |
|  |  |  | **beta** | **se** | ***P*-value** | **Outcome** | **beta** | **se** | ***P*-value** |
| rs10132280 | A | C | -0.0289 | 0.004 | 4.96E-11 | RA | -0.01005 | 0.014559 | 0.49 |
| rs11209963 | G | A | 0.0434 | 0.005 | 3.37E-17 | RA | 0.030459 | 0.025375 | 0.23 |
| rs11604680 | G | A | 0.0277 | 0.004 | 1.00E-10 | RA | 0.040822 | 0.020552 | 0.0470002 |
| rs11676272 | G | A | 0.026 | 0.005 | 5.23E-09 | RA | 0.040822 | 0.01426 | 0.00420001 |
| rs1222069 | C | A | -0.0243 | 0.004 | 1.05E-08 | RA | -0.00995 | 0.029984 | 0.74 |
| rs12286929 | G | A | 0.0222 | 0.004 | 3.55E-08 | RA | 0.01005 | 0.016813 | 0.55 |
| rs12429545 | A | G | 0.0348 | 0.006 | 1.51E-08 | RA | -0.04082 | 0.030447 | 0.18 |
| rs13107325 | T | C | 0.0525 | 0.009 | 2.72E-09 | RA | -0.03046 | 0.043125 | 0.48 |
| rs13130484 | T | C | 0.0407 | 0.004 | 6.05E-23 | RA | 0.029559 | 0.020534 | 0.15 |
| rs1421085 | C | T | 0.084 | 0.004 | 4.11E-94 | RA | -0.00995 | 0.02335 | 0.67 |
| rs1528435 | T | C | 0.0229 | 0.004 | 4.32E-08 | RA | -0.0202 | 0.019093 | 0.29 |
| rs16907751 | T | C | -0.0474 | 0.009 | 3.89E-08 | RA | 0.00995 | 0.049285 | 0.84 |
| rs17094222 | C | T | 0.0307 | 0.005 | 8.02E-10 | RA | 0.020203 | 0.027344 | 0.46 |
| rs17109256 | A | G | 0.0355 | 0.005 | 7.14E-13 | RA | 0.029559 | 0.019012 | 0.12 |
| rs2176040 | G | A | -0.0239 | 0.004 | 9.99E-09 | RA | -0.0198 | 0.017952 | 0.27 |
| rs2206277 | T | C | 0.0448 | 0.005 | 1.69E-17 | RA | 0.019803 | 0.020754 | 0.34 |
| rs253414 | T | C | 0.027 | 0.005 | 3.92E-09 | RA | 0.029559 | 0.024625 | 0.23 |
| rs2820315 | T | C | 0.0253 | 0.004 | 6.63E-09 | RA | 0.029559 | 0.022046 | 0.18 |
| rs3888190 | A | C | 0.035 | 0.004 | 1.32E-17 | RA | 0.058269 | 0.016661 | 0.00047 |
| rs4776970 | T | A | -0.0282 | 0.004 | 1.89E-11 | RA | 0.020203 | 0.023013 | 0.38 |
| rs492400 | T | C | -0.0238 | 0.004 | 6.78E-09 | RA | -0.0202 | 0.017562 | 0.25 |
| rs543874 | G | A | 0.0341 | 0.005 | 2.73E-11 | RA | -0.03922 | 0.024541 | 0.11 |
| rs6265 | T | C | -0.0466 | 0.005 | 1.09E-19 | RA | 0.019803 | 0.027409 | 0.47 |
| rs6567160 | C | T | 0.0546 | 0.005 | 4.55E-30 | RA | -0.00995 | 0.024124 | 0.68 |
| rs6734363 | A | G | 0.0524 | 0.005 | 2.01E-22 | RA | -0.01005 | 0.019704 | 0.61 |
| rs7138803 | A | G | 0.0273 | 0.004 | 5.90E-11 | RA | -0.0202 | 0.024005 | 0.4 |
| rs7599312 | A | G | -0.0256 | 0.005 | 8.68E-09 | RA | -0.03046 | 0.019059 | 0.11 |
| rs7903146 | T | C | -0.0294 | 0.005 | 3.89E-11 | RA | -0.05129 | 0.019152 | 0.00739997 |
| rs9816226 | T | A | 0.0394 | 0.005 | 3.94E-13 | RA | -0.01005 | 0.026083 | 0.7 |
| rs10132280 | A | C | -0.0289 | 0.004 | 4.96E-11 | MS | -0.01272 | 0.017804 | 0.475 |
| rs11209963 | G | A | 0.0434 | 0.005 | 3.37E-17 | MS | 0.012579 | 0.021017 | 0.5495 |
| rs11604680 | G | A | 0.0277 | 0.004 | 1.00E-10 | MS | -0.01806 | 0.017623 | 0.3054 |
| rs11676272 | G | A | 0.026 | 0.005 | 5.23E-09 | MS | 0.020305 | 0.016619 | 0.2218 |
| rs12286929 | G | A | 0.0222 | 0.004 | 3.55E-08 | MS | 0.039885 | 0.016252 | 0.0141198 |
| rs12429545 | A | G | 0.0348 | 0.006 | 1.51E-08 | MS | 0.008637 | 0.024731 | 0.726901 |
| rs13107325 | T | C | 0.0525 | 0.009 | 2.72E-09 | MS | 0.113505 | 0.037885 | 0.00273502 |
| rs13130484 | T | C | 0.0407 | 0.004 | 6.05E-23 | MS | 0.025318 | 0.016477 | 0.1244 |
| rs1421085 | C | T | 0.084 | 0.004 | 4.11E-94 | MS | 0.031491 | 0.016436 | 0.0553694 |
| rs1528435 | T | C | 0.0229 | 0.004 | 4.32E-08 | MS | 0.006678 | 0.016938 | 0.6934 |
| rs16907751 | T | C | -0.0474 | 0.009 | 3.89E-08 | MS | 0.001001 | 0.026253 | 0.9696 |
| rs17094222 | C | T | 0.0307 | 0.005 | 8.02E-10 | MS | -0.00469 | 0.020795 | 0.8216 |
| rs17109256 | A | G | 0.0355 | 0.005 | 7.14E-13 | MS | 0.027166 | 0.019825 | 0.1706 |
| rs2176040 | G | A | -0.0239 | 0.004 | 9.99E-09 | MS | 0.052592 | 0.016947 | 0.00191298 |
| rs2206277 | T | C | 0.0448 | 0.005 | 1.69E-17 | MS | -0.01064 | 0.021276 | 0.6169 |
| rs253414 | T | C | 0.027 | 0.005 | 3.92E-09 | MS | -0.03077 | 0.017196 | 0.0735699 |
| rs2820315 | T | C | 0.0253 | 0.004 | 6.63E-09 | MS | -0.00995 | 0.017962 | 0.579599 |
| rs3888190 | A | C | 0.035 | 0.004 | 1.32E-17 | MS | 0.018164 | 0.017234 | 0.2919 |
| rs4776970 | T | A | -0.0282 | 0.004 | 1.89E-11 | MS | -0.0009 | 0.016699 | 0.957 |
| rs492400 | T | C | -0.0238 | 0.004 | 6.78E-09 | MS | -0.02102 | 0.016405 | 0.2001 |
| rs543874 | G | A | 0.0341 | 0.005 | 2.73E-11 | MS | 0.009554 | 0.020331 | 0.6384 |
| rs6265 | T | C | -0.0466 | 0.005 | 1.09E-19 | MS | -0.00723 | 0.020611 | 0.7259 |
| rs6567160 | C | T | 0.0546 | 0.005 | 4.55E-30 | MS | 0.002002 | 0.018913 | 0.9157 |
| rs6734363 | A | G | 0.0524 | 0.005 | 2.01E-22 | MS | 0.00896 | 0.021446 | 0.6761 |
| rs7138803 | A | G | 0.0273 | 0.004 | 5.90E-11 | MS | 0.04051 | 0.016867 | 0.01632 |
| rs7599312 | A | G | -0.0256 | 0.005 | 8.68E-09 | MS | -0.01361 | 0.018278 | 0.4566 |
| rs7903146 | T | C | -0.0294 | 0.005 | 3.89E-11 | MS | 0.032833 | 0.018046 | 0.0688399 |
| rs9816226 | T | A | 0.0394 | 0.005 | 3.94E-13 | MS | -0.01025 | 0.021345 | 0.630999 |

Abbreviations: MCP, multisite chronic pain; EA, Effect allele; OA, other allele; beta, estimate coefficient; se, standard error of coefficient estimate; MS, multiple sclerosis; RA, rheumatoid arthritis.

## Supplementary Table S9 Harmonies data for MCP and Smoking initiation.

| **SNP** | **EA** | **OA** | **Exposure (MCP)** | | | **Outcome (Smoking)** | | |
| --- | --- | --- | --- | --- | --- | --- | --- | --- |
|  |  |  | **beta** | **se** | ***P*-value** | **beta** | **se** | ***P*-value** |
| rs10259354 | G | A | 0.0147 | 0.0026 | 3.00E-08 | 0.0180954 | 0.00384053 | 2.50E-06 |
| rs10888692 | C | G | -0.014 | 0.0025 | 5.30E-09 | 0.0008709 | 0.00361001 | 0.809 |
| rs10992729 | C | T | 0.0158 | 0.0026 | 1.10E-09 | -0.0101483 | 0.00376381 | 0.00701 |
| rs11079993 | G | T | -0.017 | 0.0025 | 5.70E-12 | -0.0164291 | 0.00366451 | 7.26E-06 |
| rs11599236 | T | C | 0.0138 | 0.0025 | 3.30E-08 | 0.0149909 | 0.00361462 | 3.38E-05 |
| rs11751591 | G | A | 0.0214 | 0.0034 | 2.70E-10 | 0.00653175 | 0.0051638 | 0.206 |
| rs11786084 | G | A | -0.015 | 0.0026 | 2.30E-08 | 0.00629612 | 0.00376939 | 0.094999 |
| rs11871043 | T | C | 0.0149 | 0.0025 | 1.70E-09 | 0.00860436 | 0.00360081 | 0.0169 |
| rs12071912 | C | T | -0.015 | 0.0026 | 5.30E-09 | 0.00077317 | 0.00383716 | 0.84 |
| rs12435797 | G | T | -0.017 | 0.0031 | 3.70E-08 | -0.003384 | 0.00453827 | 0.456 |
| rs12765185 | T | A | -0.015 | 0.0027 | 3.90E-08 | 0.011479 | 0.00402339 | 0.00434 |
| rs13135092 | A | G | -0.033 | 0.0044 | 1.50E-13 | 0.0147311 | 0.00689092 | 0.0325 |
| rs13136239 | G | A | 0.0141 | 0.0026 | 3.60E-08 | 0.0225635 | 0.00373473 | 1.50E-09 |
| rs1443914 | T | C | 0.0162 | 0.0024 | 2.80E-11 | 0.00774504 | 0.00356117 | 0.0296 |
| rs17474406 | G | A | -0.049 | 0.0088 | 2.40E-08 | 0.0109014 | 0.0119876 | 0.363 |
| rs1946247 | T | G | -0.019 | 0.0035 | 4.90E-08 | -0.0172747 | 0.00513892 | 0.00076 |
| rs197422 | C | A | -0.015 | 0.0025 | 2.00E-09 | -0.0151014 | 0.00366263 | 3.65E-05 |
| rs2006281 | C | T | 0.0135 | 0.0024 | 3.40E-08 | 0.0081667 | 0.00356085 | 0.0219 |
| rs2386584 | T | G | -0.017 | 0.0025 | 2.80E-11 | -0.0121293 | 0.00365712 | 0.000914 |
| rs2424248 | G | A | 0.023 | 0.0037 | 3.70E-10 | 0.00690247 | 0.00535735 | 0.198 |
| rs28428925 | G | A | -0.021 | 0.0035 | 1.40E-09 | -0.0011241 | 0.00523535 | 0.83 |
| rs285026 | G | T | -0.014 | 0.0025 | 1.90E-08 | -0.0020734 | 0.00358234 | 0.562999 |
| rs34811474 | G | A | 0.0192 | 0.0029 | 2.70E-11 | 0.00447835 | 0.00425066 | 0.291 |
| rs4852567 | A | G | 0.0149 | 0.0027 | 4.30E-08 | 0.0128086 | 0.00393413 | 0.00116 |
| rs59898460 | T | C | 0.0169 | 0.0025 | 9.20E-12 | 0.00481017 | 0.00364658 | 0.187 |
| rs61883178 | C | A | -0.021 | 0.0033 | 2.00E-10 | -0.0021077 | 0.00487412 | 0.665001 |
| rs6478241 | A | G | 0.0149 | 0.0025 | 3.10E-09 | 0.0117495 | 0.00369707 | 0.00146 |
| rs6770476 | C | T | -0.015 | 0.0027 | 9.40E-09 | 0.00477121 | 0.00392191 | 0.223 |
| rs6869446 | T | C | -0.014 | 0.0025 | 9.50E-09 | -0.0054898 | 0.00365177 | 0.132 |
| rs6907508 | A | G | -0.022 | 0.0038 | 1.10E-08 | -0.0139083 | 0.00551069 | 0.0116 |
| rs6926377 | A | C | -0.016 | 0.0027 | 7.90E-09 | -0.0049353 | 0.00391392 | 0.207 |
| rs6966540 | T | C | -0.014 | 0.0025 | 3.30E-08 | 0.0032069 | 0.00367615 | 0.383 |
| rs73581580 | G | A | -0.028 | 0.0037 | 5.30E-14 | -0.0062284 | 0.0053213 | 0.243 |
| rs7628207 | T | C | 0.0195 | 0.0032 | 8.40E-10 | 0.0254125 | 0.00471088 | 6.97E-08 |
| rs7798894 | A | T | 0.0153 | 0.0027 | 1.60E-08 | 0.00676586 | 0.00392595 | 0.084901 |

Abbreviation: MCP, multisite chronic pain; EA, Effect allele; OA, other allele; beta, estimate coefficient; se, standard error of coefficient estimate; MS, multiple sclerosis; RA, rheumatoid arthritis.

## Supplementary Table S10 Harmonies data for Smoking initiation and MS/RA.

| **SNP** | **EA** | **OA** | **Exposure (Smoking)** | | | **Outcome (MS/RA)** | | | |
| --- | --- | --- | --- | --- | --- | --- | --- | --- | --- |
|  |  |  | **beta** | **se** | **P** | **Outcome** | **beta** | **se** | **P** |
| rs10001365 | A | G | -0.025 | 0.00364 | 6.65E-12 | RA | 0.00995033 | 0.028831 | 0.73 |
| rs10114490 | A | G | -0.026 | 0.00453 | 1.81E-08 | RA | -0.0100503 | 0.0302855 | 0.74 |
| rs10159545 | G | C | 0.0263 | 0.00373 | 1.84E-12 | RA | -0.0304592 | 0.0211591 | 0.15 |
| rs10233018 | G | A | 0.0271 | 0.00356 | 2.75E-14 | RA | 0.0304592 | 0.0195908 | 0.12 |
| rs10260968 | A | G | -0.02 | 0.00361 | 1.75E-08 | RA | -0.0304592 | 0.0149786 | 0.042 |
| rs10279261 | A | G | -0.021 | 0.00366 | 5.00E-09 | RA | -0.0304592 | 0.022718 | 0.18 |
| rs10498846 | T | C | 0.0206 | 0.00356 | 6.62E-09 | RA | -0.0100503 | 0.0221489 | 0.649999 |
| rs1050847 | T | C | -0.022 | 0.00359 | 1.67E-09 | RA | -0.0202027 | 0.0207396 | 0.33 |
| rs10905461 | C | T | -0.024 | 0.00415 | 7.35E-09 | RA | 0.00995033 | 0.0170721 | 0.56 |
| rs11057005 | G | A | -0.021 | 0.00358 | 4.85E-09 | RA | -0.0099503 | 0.0267586 | 0.709999 |
| rs11078713 | G | A | -0.02 | 0.00361 | 2.23E-08 | RA | 0.0100503 | 0.0152546 | 0.51 |
| rs1154693 | G | A | 0.0326 | 0.00491 | 3.12E-11 | RA | 0.0618754 | 0.0218349 | 0.0046 |
| rs1160685 | G | C | 0.0208 | 0.00359 | 7.20E-09 | RA | -0.0100503 | 0.0197037 | 0.61 |
| rs11658881 | G | A | 0.0201 | 0.00361 | 2.43E-08 | RA | -0.0295588 | 0.0210372 | 0.16 |
| rs11712680 | C | A | -0.027 | 0.00458 | 3.51E-09 | RA | -0.0295588 | 0.0285197 | 0.3 |
| rs117143374 | C | T | 0.0293 | 0.00527 | 2.76E-08 | RA | 0.0512933 | 0.0302544 | 0.09 |
| rs12042107 | C | T | -0.022 | 0.00357 | 4.22E-10 | RA | 0.0100503 | 0.0142295 | 0.48 |
| rs12112638 | G | A | -0.025 | 0.00404 | 1.34E-09 | RA | 0.0100503 | 0.0396702 | 0.8 |
| rs12186738 | T | G | -0.033 | 0.00502 | 3.42E-11 | RA | -0.0100503 | 0.0291208 | 0.73 |
| rs12333760 | C | T | -0.029 | 0.0048 | 1.44E-09 | RA | 0.0725707 | 0.0264629 | 0.0061 |
| rs12356821 | C | G | 0.0394 | 0.00505 | 6.27E-15 | RA | 0.040822 | 0.0243762 | 0.094001 |
| rs12441907 | A | C | -0.029 | 0.00452 | 1.06E-10 | RA | 0.0392207 | 0.025226 | 0.12 |
| rs12474587 | T | G | 0.0276 | 0.00358 | 1.25E-14 | RA | -0.0099503 | 0.0258235 | 0.7 |
| rs12545053 | G | A | 0.0203 | 0.00364 | 2.43E-08 | RA | 0.0618754 | 0.0193684 | 0.0014 |
| rs12632110 | G | A | -0.023 | 0.00375 | 4.78E-10 | RA | 0.0100503 | 0.0136027 | 0.46 |
| rs13030994 | A | G | 0.0361 | 0.00356 | 3.56E-24 | RA | 0.0198026 | 0.0256447 | 0.44 |
| rs13145728 | C | G | -0.023 | 0.00366 | 2.14E-10 | RA | -0.0099503 | 0.0206555 | 0.630001 |
| rs13261666 | T | G | -0.027 | 0.00356 | 3.90E-14 | RA | -0.0512933 | 0.0181881 | 0.0048 |
| rs134529 | C | T | -0.02 | 0.00366 | 4.85E-08 | RA | 0.0202027 | 0.0175622 | 0.25 |
| rs1385108 | T | C | 0.0247 | 0.00416 | 3.00E-09 | RA | 0.0295588 | 0.0173259 | 0.088 |
| rs1435741 | A | G | 0.0294 | 0.00359 | 2.64E-16 | RA | 0.0392207 | 0.0179624 | 0.029 |
| rs1445649 | C | T | 0.024 | 0.00356 | 1.68E-11 | RA | -0.0099503 | 0.0325727 | 0.760001 |
| rs1555445 | T | A | 0.0226 | 0.00382 | 3.65E-09 | RA | -0.0295588 | 0.0190116 | 0.12 |
| rs1565735 | A | T | -0.038 | 0.00446 | 3.42E-17 | RA | -0.0304592 | 0.0264782 | 0.25 |
| rs1869243 | C | T | 0.0197 | 0.00356 | 2.97E-08 | RA | 0.0202027 | 0.0175622 | 0.25 |
| rs1899896 | T | C | 0.0264 | 0.00389 | 1.04E-11 | RA | 0.0295588 | 0.024625 | 0.23 |
| rs1971318 | T | C | 0.0285 | 0.00493 | 7.06E-09 | RA | 0.0198026 | 0.0286866 | 0.49 |
| rs2046850 | T | C | -0.025 | 0.00448 | 3.03E-08 | RA | 0.0198026 | 0.0286866 | 0.49 |
| rs2050586 | C | G | -0.021 | 0.00371 | 3.00E-08 | RA | 0.0304592 | 0.0221973 | 0.17 |
| rs2107300 | G | C | -0.027 | 0.00493 | 3.27E-08 | RA | -0.0202027 | 0.027963 | 0.47 |
| rs2140114 | T | C | -0.023 | 0.00373 | 4.70E-10 | RA | -0.0100503 | 0.0214889 | 0.64 |
| rs2186122 | T | A | 0.0261 | 0.00359 | 3.61E-13 | RA | 0.0202027 | 0.024521 | 0.41 |
| rs222449 | T | A | -0.025 | 0.00443 | 1.08E-08 | RA | 0.0100503 | 0.0202689 | 0.62 |
| rs2378662 | A | G | 0.0209 | 0.00357 | 4.16E-09 | RA | 0.0100503 | 0.0202689 | 0.62 |
| rs240963 | C | T | -0.041 | 0.00484 | 2.16E-17 | RA | 0.0202027 | 0.0216167 | 0.35 |
| rs2631024 | G | A | -0.023 | 0.00403 | 1.18E-08 | RA | -0.0099503 | 0.0392755 | 0.8 |
| rs266047 | A | G | -0.031 | 0.00374 | 3.36E-16 | RA | -0.0202027 | 0.0187007 | 0.28 |
| rs3001723 | A | G | 0.0335 | 0.0039 | 8.12E-18 | RA | 0.0487902 | 0.0166573 | 0.0034 |
| rs301805 | G | T | 0.0215 | 0.00361 | 2.80E-09 | RA | 0.0304592 | 0.0178537 | 0.088 |
| rs35702515 | T | G | 0.0252 | 0.00423 | 2.43E-09 | RA | -0.0202027 | 0.0321696 | 0.53 |
| rs3800227 | G | A | 0.0228 | 0.00406 | 1.93E-08 | RA | -0.0100503 | 0.0418022 | 0.81 |
| rs3904512 | A | G | -0.021 | 0.00358 | 3.23E-09 | RA | 0.00995033 | 0.0249472 | 0.69 |
| rs4352629 | T | C | -0.028 | 0.00357 | 1.22E-14 | RA | 0.00995033 | 0.0154665 | 0.52 |
| rs4523689 | G | A | -0.021 | 0.00364 | 1.55E-08 | RA | -0.0099503 | 0.0154665 | 0.52 |
| rs4543592 | C | T | 0.0219 | 0.00356 | 7.46E-10 | RA | 0.0304592 | 0.0171661 | 0.075999 |
| rs4674993 | G | A | -0.025 | 0.00444 | 1.32E-08 | RA | -0.0392207 | 0.0224772 | 0.081001 |
| rs4759228 | C | G | -0.022 | 0.00393 | 3.58E-08 | RA | -0.0392207 | 0.0192871 | 0.042 |
| rs4781977 | C | T | -0.024 | 0.00436 | 4.54E-08 | RA | -0.0099503 | 0.0189747 | 0.6 |
| rs4785836 | C | T | -0.02 | 0.00366 | 2.26E-08 | RA | -0.0392207 | 0.0218 | 0.072 |
| rs578584 | T | A | 0.0287 | 0.0036 | 1.50E-15 | RA | 0.040822 | 0.0210083 | 0.052 |
| rs6265 | T | C | -0.032 | 0.00458 | 3.77E-12 | RA | 0.0198026 | 0.0274093 | 0.47 |
| rs6433897 | C | T | 0.0224 | 0.00406 | 3.16E-08 | RA | 0.0100503 | 0.0176926 | 0.57 |
| rs6508144 | G | C | -0.021 | 0.00359 | 7.97E-09 | RA | -0.0100503 | 0.0152546 | 0.51 |
| rs66680800 | T | G | -0.02 | 0.00365 | 2.83E-08 | RA | -0.0304592 | 0.022718 | 0.18 |
| rs6669839 | T | C | 0.026 | 0.0044 | 3.36E-09 | RA | 0.0295588 | 0.0279352 | 0.29 |
| rs6728726 | C | T | 0.0354 | 0.00473 | 6.73E-14 | RA | -0.0198026 | 0.0262142 | 0.450001 |
| rs6788098 | T | A | -0.031 | 0.00369 | 1.91E-17 | RA | -0.0295588 | 0.022554 | 0.19 |
| rs6893752 | G | A | -0.024 | 0.00407 | 3.25E-09 | RA | 0.040822 | 0.0245979 | 0.097 |
| rs7197072 | T | C | -0.025 | 0.00417 | 2.77E-09 | RA | 0.00995033 | 0.0463449 | 0.83 |
| rs7224742 | T | C | -0.021 | 0.00366 | 1.43E-08 | RA | 0.0392207 | 0.0181824 | 0.031 |
| rs72789632 | T | C | -0.033 | 0.00529 | 5.02E-10 | RA | 0.0295588 | 0.026242 | 0.26 |
| rs72896886 | C | G | -0.027 | 0.00484 | 2.75E-08 | RA | -0.0099503 | 0.0299841 | 0.74 |
| rs7555507 | T | C | -0.024 | 0.00356 | 1.14E-11 | RA | -0.0100503 | 0.0136027 | 0.46 |
| rs7585579 | G | C | 0.0224 | 0.00373 | 1.88E-09 | RA | 0.0295588 | 0.0173259 | 0.088 |
| rs76214862 | C | A | -0.025 | 0.00455 | 3.99E-08 | RA | -0.0198026 | 0.0268021 | 0.46 |
| rs7921378 | C | G | -0.025 | 0.00356 | 8.26E-13 | RA | -0.0099503 | 0.0147524 | 0.5 |
| rs7929518 | G | A | 0.0242 | 0.00428 | 1.56E-08 | RA | 0.0202027 | 0.0194925 | 0.3 |
| rs7938812 | G | T | 0.0438 | 0.00364 | 2.71E-33 | RA | 0.0202027 | 0.017194 | 0.24 |
| rs7969559 | G | A | -0.024 | 0.00396 | 7.31E-10 | RA | -0.0198026 | 0.019913 | 0.32 |
| rs9401770 | A | G | 0.0277 | 0.00399 | 3.47E-12 | RA | -0.0100503 | 0.025198 | 0.69 |
| rs9423279 | G | C | -0.021 | 0.00371 | 3.21E-08 | RA | -0.0512933 | 0.0465008 | 0.27 |
| rs9540729 | T | A | -0.02 | 0.00356 | 3.82E-08 | RA | -0.0582689 | 0.0204599 | 0.0044 |
| rs962625 | G | A | 0.0237 | 0.00404 | 4.37E-09 | RA | 0.040822 | 0.0203636 | 0.045 |
| rs9835772 | T | A | 0.024 | 0.00414 | 6.32E-09 | RA | 0.0100503 | 0.0142295 | 0.48 |
| rs993700 | C | T | -0.026 | 0.00429 | 1.53E-09 | RA | -0.0295588 | 0.0215411 | 0.17 |
| rs10001365 | A | G | -0.025 | 0.00364 | 6.65E-12 | MS | -0.005087 | 0.0167244 | 0.761001 |
| rs10114490 | A | G | -0.026 | 0.00453 | 1.81E-08 | MS | 0.0150121 | 0.0206621 | 0.4675 |
| rs10159545 | G | C | 0.0263 | 0.00373 | 1.84E-12 | MS | 0.0337636 | 0.0173855 | 0.05213 |
| rs10233018 | G | A | 0.0271 | 0.00356 | 2.75E-14 | MS | -0.0068763 | 0.0164587 | 0.6761 |
| rs10260968 | A | G | -0.02 | 0.00361 | 1.75E-08 | MS | -0.0124775 | 0.0165796 | 0.4517 |
| rs10279261 | A | G | -0.021 | 0.00366 | 5.00E-09 | MS | 0.013592 | 0.0167392 | 0.4168 |
| rs10498846 | T | C | 0.0206 | 0.00356 | 6.62E-09 | MS | 0.00975229 | 0.0165632 | 0.556 |
| rs1050847 | T | C | -0.022 | 0.00359 | 1.67E-09 | MS | -0.0254204 | 0.0168634 | 0.1317 |
| rs10905461 | C | T | -0.024 | 0.00415 | 7.35E-09 | MS | -0.0174514 | 0.0186922 | 0.3505 |
| rs11057005 | G | A | -0.021 | 0.00358 | 4.85E-09 | MS | -0.0218367 | 0.0163842 | 0.1826 |
| rs11078713 | G | A | -0.02 | 0.00361 | 2.23E-08 | MS | -0.0209172 | 0.0167082 | 0.2106 |
| rs1154693 | G | A | 0.0326 | 0.00491 | 3.12E-11 | MS | -0.0151136 | 0.0230487 | 0.512 |
| rs1160685 | G | C | 0.0208 | 0.00359 | 7.20E-09 | MS | -0.0165621 | 0.0163644 | 0.3115 |
| rs11658881 | G | A | 0.0201 | 0.00361 | 2.43E-08 | MS | -0.0073268 | 0.0164075 | 0.6552 |
| rs11712680 | C | A | -0.027 | 0.00458 | 3.51E-09 | MS | 0.0134906 | 0.0216075 | 0.5324 |
| rs117143374 | C | T | 0.0293 | 0.00527 | 2.76E-08 | MS | 0.00954541 | 0.0256511 | 0.7098 |
| rs11872397 | A | G | -0.025 | 0.00409 | 1.43E-09 | MS | 0.0119286 | 0.0190824 | 0.5319 |
| rs12025237 | C | A | -0.033 | 0.00534 | 6.52E-10 | MS | 0.0319036 | 0.0248557 | 0.1993 |
| rs12042107 | C | T | -0.022 | 0.00357 | 4.22E-10 | MS | -0.0228593 | 0.0181196 | 0.2071 |
| rs12112638 | G | A | -0.025 | 0.00404 | 1.34E-09 | MS | 0.0036065 | 0.0183395 | 0.8441 |
| rs12186738 | T | G | -0.033 | 0.00502 | 3.42E-11 | MS | -0.0194104 | 0.0226614 | 0.3917 |
| rs12333760 | C | T | -0.029 | 0.0048 | 1.44E-09 | MS | 0.0344014 | 0.0220366 | 0.1185 |
| rs12356821 | C | G | 0.0394 | 0.00505 | 6.27E-15 | MS | 0.00150113 | 0.0347059 | 0.9655 |
| rs12441907 | A | C | -0.029 | 0.00452 | 1.06E-10 | MS | 0.00030005 | 0.0193058 | 0.9876 |
| rs12474587 | T | G | 0.0276 | 0.00358 | 1.25E-14 | MS | -0.0119286 | 0.016604 | 0.4725 |
| rs12545053 | G | A | 0.0203 | 0.00364 | 2.43E-08 | MS | -0.0028958 | 0.0167694 | 0.8629 |
| rs12632110 | G | A | -0.023 | 0.00375 | 4.78E-10 | MS | 0.00995033 | 0.0172476 | 0.564 |
| rs13030994 | A | G | 0.0361 | 0.00356 | 3.56E-24 | MS | 0.0041912 | 0.0162037 | 0.7959 |
| rs13145728 | C | G | -0.023 | 0.00366 | 2.14E-10 | MS | -0.0464064 | 0.0168867 | 0.005994 |
| rs13261666 | T | G | -0.027 | 0.00356 | 3.90E-14 | MS | 0.0113643 | 0.0162078 | 0.4832 |
| rs134529 | C | T | -0.02 | 0.00366 | 4.85E-08 | MS | 0.0334348 | 0.0167937 | 0.04649 |
| rs1385108 | T | C | 0.0247 | 0.00416 | 3.00E-09 | MS | -0.0007997 | 0.0180692 | 0.9647 |
| rs1435741 | A | G | 0.0294 | 0.00359 | 2.64E-16 | MS | -0.001401 | 0.0167394 | 0.9333 |
| rs1445649 | C | T | 0.024 | 0.00356 | 1.68E-11 | MS | 0.0174469 | 0.0162778 | 0.2838 |
| rs1555445 | T | A | 0.0226 | 0.00382 | 3.65E-09 | MS | 0.0491902 | 0.018298 | 0.007182 |
| rs1565735 | A | T | -0.038 | 0.00446 | 3.42E-17 | MS | -0.0541093 | 0.0204744 | 0.008223 |
| rs1869243 | C | T | 0.0197 | 0.00356 | 2.97E-08 | MS | 0.0105555 | 0.0163445 | 0.5184 |
| rs1899896 | T | C | 0.0264 | 0.00389 | 1.04E-11 | MS | 0.0284903 | 0.018131 | 0.1161 |
| rs1971318 | T | C | 0.0285 | 0.00493 | 7.06E-09 | MS | 0.0150121 | 0.0227374 | 0.5091 |
| rs2046850 | T | C | -0.025 | 0.00448 | 3.03E-08 | MS | 0.00521357 | 0.0206844 | 0.801 |
| rs2050586 | C | G | -0.021 | 0.00371 | 3.00E-08 | MS | 0.00478852 | 0.0169458 | 0.777499 |
| rs2107300 | G | C | -0.027 | 0.00493 | 3.27E-08 | MS | 0.00410843 | 0.0234802 | 0.8611 |
| rs2140114 | T | C | -0.023 | 0.00373 | 4.70E-10 | MS | 0.0268367 | 0.0163492 | 0.1007 |
| rs2186122 | T | A | 0.0261 | 0.00359 | 3.61E-13 | MS | -0.005286 | 0.0163858 | 0.747001 |
| rs222449 | T | A | -0.025 | 0.00443 | 1.08E-08 | MS | -0.0054146 | 0.0200099 | 0.7867 |
| rs2378662 | A | G | 0.0209 | 0.00357 | 4.16E-09 | MS | 0.0201007 | 0.0180823 | 0.2663 |
| rs240963 | C | T | -0.041 | 0.00484 | 2.16E-17 | MS | -0.0051131 | 0.0222566 | 0.8183 |
| rs2631024 | G | A | -0.023 | 0.00403 | 1.18E-08 | MS | 0.00461061 | 0.0186754 | 0.805 |
| rs266047 | A | G | -0.031 | 0.00374 | 3.36E-16 | MS | -0.0046106 | 0.0165762 | 0.780899 |
| rs3001723 | A | G | 0.0335 | 0.0039 | 8.12E-18 | MS | 0.00682323 | 0.0175969 | 0.6982 |
| rs301805 | G | T | 0.0215 | 0.00361 | 2.80E-09 | MS | -0.018979 | 0.0165473 | 0.2514 |
| rs35702515 | T | G | 0.0252 | 0.00423 | 2.43E-09 | MS | -0.0560022 | 0.0235675 | 0.01749 |
| rs3800227 | G | A | 0.0228 | 0.00406 | 1.93E-08 | MS | -0.0049875 | 0.0183697 | 0.786001 |
| rs3801289 | C | A | -0.022 | 0.00374 | 3.74E-09 | MS | -0.0196065 | 0.0202738 | 0.3335 |
| rs3904512 | A | G | -0.021 | 0.00358 | 3.23E-09 | MS | -0.005286 | 0.0163992 | 0.7472 |
| rs4044321 | G | A | -0.028 | 0.00371 | 6.08E-14 | MS | 0.0142973 | 0.016995 | 0.4002 |
| rs4236259 | G | T | -0.025 | 0.00356 | 3.35E-12 | MS | -0.0228372 | 0.0184503 | 0.2158 |
| rs4352629 | T | C | -0.028 | 0.00357 | 1.22E-14 | MS | 0.00060018 | 0.0158006 | 0.9697 |
| rs4523689 | G | A | -0.021 | 0.00364 | 1.55E-08 | MS | -0.0178585 | 0.0166515 | 0.2835 |
| rs4543592 | C | T | 0.0219 | 0.00356 | 7.46E-10 | MS | 0.0140015 | 0.0164924 | 0.3959 |
| rs4674993 | G | A | -0.025 | 0.00444 | 1.32E-08 | MS | -0.0244975 | 0.0205929 | 0.2342 |
| rs4759228 | C | G | -0.022 | 0.00393 | 3.58E-08 | MS | 0.00767051 | 0.0201252 | 0.7031 |
| rs4781977 | C | T | -0.024 | 0.00436 | 4.54E-08 | MS | -0.0033942 | 0.020501 | 0.8685 |
| rs4785836 | C | T | -0.02 | 0.00366 | 2.26E-08 | MS | 0.00712533 | 0.0170436 | 0.675899 |
| rs578584 | T | A | 0.0287 | 0.0036 | 1.50E-15 | MS | -0.0265493 | 0.0167054 | 0.112 |
| rs6265 | T | C | -0.032 | 0.00458 | 3.77E-12 | MS | -0.0072261 | 0.0206114 | 0.7259 |
| rs6433897 | C | T | 0.0224 | 0.00406 | 3.16E-08 | MS | -0.0377019 | 0.0185492 | 0.0421 |
| rs6508144 | G | C | -0.021 | 0.00359 | 7.97E-09 | MS | 0.00149888 | 0.0162039 | 0.9263 |
| rs66680800 | T | G | -0.02 | 0.00365 | 2.83E-08 | MS | -0.006479 | 0.0166337 | 0.696899 |
| rs6669839 | T | C | 0.026 | 0.0044 | 3.36E-09 | MS | -0.0053855 | 0.0209891 | 0.7975 |
| rs6728726 | C | T | 0.0354 | 0.00473 | 6.73E-14 | MS | 0.0030952 | 0.0213816 | 0.8849 |
| rs6788098 | T | A | -0.031 | 0.00369 | 1.91E-17 | MS | -0.0072261 | 0.0168859 | 0.6687 |
| rs6893752 | G | A | -0.024 | 0.00407 | 3.25E-09 | MS | -0.0211215 | 0.0185711 | 0.2554 |
| rs7197072 | T | C | -0.025 | 0.00417 | 2.77E-09 | MS | -0.0398936 | 0.0197499 | 0.04339 |
| rs7224742 | T | C | -0.021 | 0.00366 | 1.43E-08 | MS | 0.00149888 | 0.0164048 | 0.9272 |
| rs72789632 | T | C | -0.033 | 0.00529 | 5.02E-10 | MS | 0.03015 | 0.0241729 | 0.2123 |
| rs72896886 | C | G | -0.027 | 0.00484 | 2.75E-08 | MS | -0.0099503 | 0.0233044 | 0.6694 |
| rs7322872 | T | C | -0.026 | 0.00433 | 3.58E-09 | MS | -0.0029042 | 0.0253248 | 0.9087 |
| rs7555507 | T | C | -0.024 | 0.00356 | 1.14E-11 | MS | -0.0041912 | 0.0163926 | 0.7982 |
| rs7585579 | G | C | 0.0224 | 0.00373 | 1.88E-09 | MS | -0.0117309 | 0.0164363 | 0.4754 |
| rs76214862 | C | A | -0.025 | 0.00455 | 3.99E-08 | MS | -0.026155 | 0.0215469 | 0.2248 |
| rs78411160 | C | A | 0.0205 | 0.00366 | 2.03E-08 | MS | 0.0151136 | 0.0165972 | 0.3625 |
| rs7921378 | C | G | -0.025 | 0.00356 | 8.26E-13 | MS | -0.0156765 | 0.0166869 | 0.3475 |
| rs7929518 | G | A | 0.0242 | 0.00428 | 1.56E-08 | MS | -0.0365603 | 0.0192242 | 0.057201 |
| rs7938812 | G | T | 0.0438 | 0.00364 | 2.71E-33 | MS | -0.0060815 | 0.0169214 | 0.7193 |
| rs7969559 | G | A | -0.024 | 0.00396 | 7.31E-10 | MS | -0.0134906 | 0.0178861 | 0.4507 |
| rs9401770 | A | G | 0.0277 | 0.00399 | 3.47E-12 | MS | 0.0180621 | 0.017965 | 0.3147 |
| rs9423279 | G | C | -0.021 | 0.00371 | 3.21E-08 | MS | -0.0463581 | 0.0305298 | 0.1289 |
| rs9540729 | T | A | -0.02 | 0.00356 | 3.82E-08 | MS | 0.0193123 | 0.016248 | 0.2346 |
| rs962625 | G | A | 0.0237 | 0.00404 | 4.37E-09 | MS | 0.00581689 | 0.0186801 | 0.755499 |
| rs9835772 | T | A | 0.024 | 0.00414 | 6.32E-09 | MS | -0.0066219 | 0.0190109 | 0.727601 |
| rs993700 | C | T | -0.026 | 0.00429 | 1.53E-09 | MS | 0.0118298 | 0.0191902 | 0.5376 |

Abbreviations: MCP, multisite chronic pain; RA, rheumatoid arthritis; MS, multiple sclerosis; EA, Effect allele; OA, other allele; beta, estimate coefficient; se, standard error of coefficient estimate.

## Supplementary Table S11 Effect estimates of multisite chronic pain on mediators and of mediators on MS/RA in two-step Mendelian randomization analyses.

| **Mediator** | **Method** | **Effect of MCP on mediators** | | **Effect of mediators on MS** | | **Effect of mediators on RA** | |
| --- | --- | --- | --- | --- | --- | --- | --- |
|  |  | **Effect estimate: OR (95% CI)** | ***P*** | **Effect estimate: OR (95% CI)** | ***P*** | **Effect estimate: OR (95% CI)** | ***P*** |
| BMI | IVW | 1.19 (1.26, 1.67) | 0.027 | 1.28 (0.99, 1.66) | 0.057 | 1.43 (1.07, 1.92) | 0.016 |
|  | MR Egger | 1.59 (0.53, 4.71) | 0.414 | 1.56 (0.79, 3.09) | 0.214 | 0.55 (0.26, 1.16) | 0.130 |
|  | Simple mode | 0.98 (0.66, 1.45) | 0.907 | 1.37 (0.89, 2.10) | 0.163 | 2.09 (0.85, 5.17) | 0.122 |
|  | Weighted median | 1.09 (0.89, 1.33) | 0.400 | 1.36 (1.01, 1.83) | 0.044 | 1.41 (0.99, 2.01) | 0.056 |
|  | Weighted mode | 0.99 (0.68, 1.42) | 0.941 | 1.39 (1.02, 1.88) | 0.046 | 0.96 (0.60, 1.54) | 0.863 |
| Smoking initiation | IVW | 1.44 (1.19, 1.73) | 0.0001 | 1.04 (0.88, 1.23) | 0.642 | 1.50 (1.17, 1.92) | 0.001 |
|  | MR Egger | 0.75 (0.33, 1.70) | 0.493 | 0.67 (0.28, 1.59) | 0.364 | 1.32 (0.39, 4.49) | 0.655 |
|  | Simple mode | 1.38 (1.00, 1.92) | 0.060 | 0.98 (0.58, 1.66) | 0.954 | 2.35 (1.00, 5.48) | 0.053 |
|  | Weighted median | 1.34 (1.15, 1.56) | 0.0001 | 1.11 (0.88, 1.39) | 0.382 | 1.59 (1.14, 2.21) | 0.007 |
|  | Weighted mode | 1.33 (1.01, 1.75) | 0.048 | 0.99 (0.60, 1.64) | 0.985 | 2.00 (0.96, 4.17) | 0.068 |

Abbreviations: MCP, multisite chronic pain; BMI, body mass index; MS, multiple sclerosis; RA, rheumatoid arthritis; IVW, inverse variance weighted; OR, odds ratio; ICI, confidence interval.

## Supplementary Table S12 Heterogeneity tests for the effect of MCP on mediators and the effect of mediators on MS/RA.

| Exposure | Outcome | Method | Q | Q_df | *P*-value |
| --- | --- | --- | --- | --- | --- |
| MCP | BMI | MR Egger | 31.093859 | 23 | 0.121 |
|  |  | IVW | 31.4669816 | 24 | 0.141 |
|  | Smoking initiation | MR Egger | 159.583058 | 32 | <0.001 |
|  |  | IVW | 172.305769 | 33 | <0.001 |
| BMI | RA | MR Egger | 35.5295727 | 25 | 0.079 |
|  |  | IVW | 45.813401 | 26 | 0.01 |
|  | MS | MR Egger | 45.5745245 | 24 | 0.005 |
|  |  | IVW | 46.2745508 | 25 | 0.006 |
| Smoking initiation | RA | MR Egger | 99.9067647 | 63 | 0.002 |
|  |  | IVW | 99.9756205 | 64 | 0.003 |
|  | MS | MR Egger | 63.228321 | 70 | 0.704 |
|  |  | IVW | 64.2709855 | 71 | 0.701 |

Abbreviations: MCP, multisite chronic pain; BMI, body mass index; MS, multiple sclerosis; RA, rheumatoid arthritis; IVW, Inverse variance weighted.

## Supplementary Table S13 Pleiotropy for the effect of MCP on mediators and the effect of mediators on MS/RA.

| **Exposure** | **Outcome** | **egger_intercept** | **se** | ***P*-value** |
| --- | --- | --- | --- | --- |
| MCP | BMI | -0.0128556 | 0.00777101 | 0.11293698 |
| MCP | Smoking initiation | 0.01136599 | 0.007116 | 0.12004058 |
| BMI | RA | 0.03648644 | 0.01356373 | 0.01254507 |
|  | MS | -0.0080433 | 0.01324743 | 0.54944777 |
| Smoking initiation | RA | 0.00333853 | 0.01602184 | 0.8356094 |
|  | MS | 0.01154341 | 0.01130477 | 0.31071869 |

Abbreviations: MCP, multisite chronic pain; BMI, body mass index; MS, multiple sclerosis; RA, rheumatoid arthritis; se, standard error of coefficient estimate.

## Supplementary Table S14 leave-one-out for the effect of MCP on BMI.

| **Exposure** | **Outcome** | **Sample size** | **SNP** | **b** | **se** | ***P*-value** |
| --- | --- | --- | --- | --- | --- | --- |
| MCP | BMI | 104447 | rs10259354 | 0.20191 | 0.074742 | 0.006904 |
| MCP | BMI | 104447 | rs10992729 | 0.188519 | 0.080468 | 0.019141 |
| MCP | BMI | 104447 | rs11079993 | 0.185539 | 0.081492 | 0.022799 |
| MCP | BMI | 104447 | rs11599236 | 0.15239 | 0.077933 | 0.050536 |
| MCP | BMI | 104447 | rs11751591 | 0.146095 | 0.076411 | 0.055881 |
| MCP | BMI | 104447 | rs11871043 | 0.204447 | 0.0785 | 0.009203 |
| MCP | BMI | 104447 | rs12435797 | 0.165474 | 0.081035 | 0.04115 |
| MCP | BMI | 104447 | rs13136239 | 0.15516 | 0.078835 | 0.049049 |
| MCP | BMI | 104447 | rs1443914 | 0.173552 | 0.082116 | 0.034559 |
| MCP | BMI | 104447 | rs1946247 | 0.182649 | 0.080783 | 0.023761 |
| MCP | BMI | 104447 | rs197422 | 0.151836 | 0.078601 | 0.053392 |
| MCP | BMI | 104447 | rs1976423 | 0.167777 | 0.081083 | 0.038526 |
| MCP | BMI | 104447 | rs2183271 | 0.161853 | 0.079503 | 0.04177 |
| MCP | BMI | 104447 | rs2386584 | 0.180085 | 0.083019 | 0.030067 |
| MCP | BMI | 104447 | rs2424248 | 0.188317 | 0.08048 | 0.019287 |
| MCP | BMI | 104447 | rs285026 | 0.184232 | 0.080677 | 0.022397 |
| MCP | BMI | 104447 | rs4852567 | 0.187916 | 0.079636 | 0.018291 |
| MCP | BMI | 104447 | rs61883178 | 0.177726 | 0.081779 | 0.029762 |
| MCP | BMI | 104447 | rs6478241 | 0.185026 | 0.080735 | 0.02192 |
| MCP | BMI | 104447 | rs6869446 | 0.177495 | 0.081371 | 0.02916 |
| MCP | BMI | 104447 | rs6907508 | 0.157794 | 0.080468 | 0.049883 |
| MCP | BMI | 104447 | rs6926377 | 0.17798 | 0.081461 | 0.0289 |
| MCP | BMI | 104447 | rs6966540 | 0.162709 | 0.080332 | 0.042821 |
| MCP | BMI | 104447 | rs7628207 | 0.146772 | 0.076765 | 0.055882 |
| MCP | BMI | 104447 | rs7798894 | 0.175926 | 0.081412 | 0.0307 |
| MCP | BMI | 104447 | All | 0.173517 | 0.078385 | 0.026853 |

Abbreviations: MCP, multisite chronic pain; BMI, body mass index; beta, estimate coefficient; se, standard error of coefficient estimate.

## Supplementary Table S15 leave-one-out for the effect of BMI on RA/MS.

| **Exposure** | **Outcome** | **Sample size** | **SNP** | **b** | **se** | ***P*-Value** |
| --- | --- | --- | --- | --- | --- | --- |
| BMI | RA | 58284 | rs10132280 | 0.361193 | 0.156408 | 0.020927 |
| BMI | RA | 58284 | rs11209963 | 0.347372 | 0.154759 | 0.024794 |
| BMI | RA | 58284 | rs11604680 | 0.33427 | 0.15031 | 0.026157 |
| BMI | RA | 58284 | rs11676272 | 0.307279 | 0.146884 | 0.03644 |
| BMI | RA | 58284 | rs1222069 | 0.360111 | 0.153088 | 0.018657 |
| BMI | RA | 58284 | rs12286929 | 0.358438 | 0.15414 | 0.02005 |
| BMI | RA | 58284 | rs12429545 | 0.386359 | 0.148396 | 0.009226 |
| BMI | RA | 58284 | rs13107325 | 0.378542 | 0.151643 | 0.01255 |
| BMI | RA | 58284 | rs13130484 | 0.341343 | 0.155451 | 0.028104 |
| BMI | RA | 58284 | rs1421085 | 0.45458 | 0.160154 | 0.004534 |
| BMI | RA | 58284 | rs1528435 | 0.383617 | 0.150013 | 0.010551 |
| BMI | RA | 58284 | rs16907751 | 0.367293 | 0.152843 | 0.016258 |
| BMI | RA | 58284 | rs17094222 | 0.355688 | 0.153494 | 0.020489 |
| BMI | RA | 58284 | rs17109256 | 0.338679 | 0.154548 | 0.028422 |
| BMI | RA | 58284 | rs2176040 | 0.34976 | 0.153524 | 0.022714 |
| BMI | RA | 58284 | rs2206277 | 0.355403 | 0.15711 | 0.023689 |
| BMI | RA | 58284 | rs253414 | 0.349154 | 0.152519 | 0.022065 |
| BMI | RA | 58284 | rs2820315 | 0.346801 | 0.152267 | 0.022752 |
| BMI | RA | 58284 | rs3888190 | 0.283195 | 0.142639 | 0.0471 |
| BMI | RA | 58284 | rs492400 | 0.348877 | 0.153502 | 0.023039 |
| BMI | RA | 58284 | rs543874 | 0.398444 | 0.146546 | 0.00655 |
| BMI | RA | 58284 | rs6265 | 0.390413 | 0.152154 | 0.010291 |
| BMI | RA | 58284 | rs6567160 | 0.398233 | 0.154857 | 0.010122 |
| BMI | RA | 58284 | rs6734363 | 0.414945 | 0.155598 | 0.007658 |
| BMI | RA | 58284 | rs7138803 | 0.378877 | 0.151022 | 0.012116 |
| BMI | RA | 58284 | rs7599312 | 0.341101 | 0.152074 | 0.024897 |
| BMI | RA | 58284 | rs7903146 | 0.317878 | 0.146709 | 0.030256 |
| BMI | RA | 58284 | All | 0.360522 | 0.149491 | 0.01588 |
| BMI | MS | 115803 | rs10132280 | 0.245245 | 0.135879 | 0.071095 |
| BMI | MS | 115803 | rs11209963 | 0.248396 | 0.137088 | 0.069995 |
| BMI | MS | 115803 | rs11604680 | 0.271404 | 0.132859 | 0.041072 |
| BMI | MS | 115803 | rs11676272 | 0.237603 | 0.13486 | 0.078094 |
| BMI | MS | 115803 | rs12286929 | 0.22256 | 0.1287 | 0.083757 |
| BMI | MS | 115803 | rs12429545 | 0.250085 | 0.135597 | 0.065137 |
| BMI | MS | 115803 | rs13107325 | 0.215056 | 0.124649 | 0.084475 |
| BMI | MS | 115803 | rs13130484 | 0.227519 | 0.136998 | 0.096764 |
| BMI | MS | 115803 | rs1421085 | 0.209656 | 0.153645 | 0.172394 |
| BMI | MS | 115803 | rs1528435 | 0.249327 | 0.135494 | 0.06575 |
| BMI | MS | 115803 | rs16907751 | 0.258583 | 0.136068 | 0.05738 |
| BMI | MS | 115803 | rs17094222 | 0.258437 | 0.135196 | 0.055931 |
| BMI | MS | 115803 | rs17109256 | 0.23411 | 0.135098 | 0.083115 |
| BMI | MS | 115803 | rs2176040 | 0.296534 | 0.116412 | 0.010857 |
| BMI | MS | 115803 | rs2206277 | 0.271162 | 0.135572 | 0.045486 |
| BMI | MS | 115803 | rs253414 | 0.282871 | 0.128557 | 0.027782 |
| BMI | MS | 115803 | rs2820315 | 0.262222 | 0.134368 | 0.050996 |
| BMI | MS | 115803 | rs3888190 | 0.239252 | 0.136545 | 0.079743 |
| BMI | MS | 115803 | rs492400 | 0.237327 | 0.13441 | 0.077448 |
| BMI | MS | 115803 | rs543874 | 0.249235 | 0.136135 | 0.067132 |
| BMI | MS | 115803 | rs6265 | 0.254822 | 0.137596 | 0.064032 |
| BMI | MS | 115803 | rs6567160 | 0.268102 | 0.139279 | 0.054238 |
| BMI | MS | 115803 | rs6734363 | 0.254729 | 0.138194 | 0.06529 |
| BMI | MS | 115803 | rs7138803 | 0.219039 | 0.129866 | 0.09167 |
| BMI | MS | 115803 | rs7599312 | 0.244785 | 0.135353 | 0.07053 |
| BMI | MS | 115803 | rs7903146 | 0.284871 | 0.128343 | 0.026446 |
| BMI | MS | 115803 | All | 0.25005 | 0.131621 | 0.057463 |

Abbreviations: MCP, multisite chronic pain; BMI, body mass index; MS, multiple sclerosis; RA, rheumatoid arthritis; beta, estimate coefficient; se, standard error of coefficient estimate.

## Supplementary Table S16 leave-one-out for the effect of MCP on Smoking.

| **Exposure** | **Outcome** | **Sample size** | **SNP** | **b** | **se** | ***P*-value** |
| --- | --- | --- | --- | --- | --- | --- |
| MCP | smoking initiation | 632802 | rs10259354 | 0.34011502 | 0.09439968 | 0.00031466 |
| MCP | smoking initiation | 632802 | rs10992729 | 0.3941889 | 0.09256158 | 2.06E-05 |
| MCP | smoking initiation | 632802 | rs11079993 | 0.33915684 | 0.09602749 | 0.00041263 |
| MCP | smoking initiation | 632802 | rs11599236 | 0.34396821 | 0.09541503 | 0.00031219 |
| MCP | smoking initiation | 632802 | rs11751591 | 0.36440606 | 0.09786872 | 0.00019654 |
| MCP | smoking initiation | 632802 | rs11786084 | 0.38354541 | 0.09490636 | 5.32E-05 |
| MCP | smoking initiation | 632802 | rs11871043 | 0.35610612 | 0.09764922 | 0.00026554 |
| MCP | smoking initiation | 632802 | rs12071912 | 0.37431387 | 0.0969793 | 0.00011351 |
| MCP | smoking initiation | 632802 | rs12435797 | 0.36695019 | 0.09753798 | 0.00016848 |
| MCP | smoking initiation | 632802 | rs12765185 | 0.39064047 | 0.09231761 | 2.32E-05 |
| MCP | smoking initiation | 632802 | rs13135092 | 0.39570072 | 0.09382635 | 2.47E-05 |
| MCP | smoking initiation | 632802 | rs13136239 | 0.33142378 | 0.09107031 | 0.00027348 |
| MCP | smoking initiation | 632802 | rs1443914 | 0.35837328 | 0.09811173 | 0.00025949 |
| MCP | smoking initiation | 632802 | rs17474406 | 0.38015245 | 0.09616036 | 7.71E-05 |
| MCP | smoking initiation | 632802 | rs1946247 | 0.34943879 | 0.09639001 | 0.00028867 |
| MCP | smoking initiation | 632802 | rs197422 | 0.34344082 | 0.09579393 | 0.00033681 |
| MCP | smoking initiation | 632802 | rs2006281 | 0.35648469 | 0.09739678 | 0.00025209 |
| MCP | smoking initiation | 632802 | rs2386584 | 0.3490752 | 0.09735736 | 0.00033643 |
| MCP | smoking initiation | 632802 | rs2424248 | 0.36470681 | 0.09797375 | 0.00019726 |
| MCP | smoking initiation | 632802 | rs28428925 | 0.37186384 | 0.09737377 | 0.00013403 |
| MCP | smoking initiation | 632802 | rs285026 | 0.36823652 | 0.09748762 | 0.00015856 |
| MCP | smoking initiation | 632802 | rs34811474 | 0.36737617 | 0.09806673 | 0.00017954 |
| MCP | smoking initiation | 632802 | rs4852567 | 0.35002909 | 0.09660491 | 0.00029086 |
| MCP | smoking initiation | 632802 | rs59898460 | 0.36565607 | 0.09822355 | 0.00019711 |
| MCP | smoking initiation | 632802 | rs61883178 | 0.37113472 | 0.09760996 | 0.0001434 |
| MCP | smoking initiation | 632802 | rs6478241 | 0.35036138 | 0.09694008 | 0.00030127 |
| MCP | smoking initiation | 632802 | rs6770476 | 0.3810438 | 0.09567964 | 6.82E-05 |
| MCP | smoking initiation | 632802 | rs6869446 | 0.36213816 | 0.09774251 | 0.00021137 |
| MCP | smoking initiation | 632802 | rs6907508 | 0.35499508 | 0.09738973 | 0.00026729 |
| MCP | smoking initiation | 632802 | rs6926377 | 0.36388251 | 0.09774673 | 0.0001971 |
| MCP | smoking initiation | 632802 | rs6966540 | 0.37766958 | 0.09616228 | 8.59E-05 |
| MCP | smoking initiation | 632802 | rs73581580 | 0.36941472 | 0.09860626 | 0.00017942 |
| MCP | smoking initiation | 632802 | rs7628207 | 0.33397598 | 0.09333983 | 0.00034615 |
| MCP | smoking initiation | 632802 | rs7798894 | 0.36050829 | 0.09768472 | 0.00022379 |
| MCP | smoking initiation | 632802 | All | 0.36265094 | 0.09495079 | 0.0001338 |

Abbreviations: MCP, multisite chronic pain; MS, multiple sclerosis; RA, rheumatoid arthritis; beta, estimate coefficient; se, standard error of coefficient estimate.

## Supplementary Table S17 leave-one-out for the effect of smoking on RA/MS.

| **Exposure** | **Outcome** | **Sample size** | **SNP** | **b** | **se** | ***P*-value** |
| --- | --- | --- | --- | --- | --- | --- |
| smoking initiation | RA | 58284 | rs10001365 | 0.41370637 | 0.12709424 | 0.00113346 |
| smoking initiation | RA | 58284 | rs10114490 | 0.40761269 | 0.12738058 | 0.00137447 |
| smoking initiation | RA | 58284 | rs10233018 | 0.39332943 | 0.1275245 | 0.00204001 |
| smoking initiation | RA | 58284 | rs10260968 | 0.38673404 | 0.12668401 | 0.00226758 |
| smoking initiation | RA | 58284 | rs10279261 | 0.39827571 | 0.12690741 | 0.00169921 |
| smoking initiation | RA | 58284 | rs10498846 | 0.41545182 | 0.12703538 | 0.0010741 |
| smoking initiation | RA | 58284 | rs1050847 | 0.40163615 | 0.1274322 | 0.00162288 |
| smoking initiation | RA | 58284 | rs10905461 | 0.42429622 | 0.12733337 | 0.00086172 |
| smoking initiation | RA | 58284 | rs11057005 | 0.40708957 | 0.1273151 | 0.00138626 |
| smoking initiation | RA | 58284 | rs11078713 | 0.42388192 | 0.12712382 | 0.00085483 |
| smoking initiation | RA | 58284 | rs1154693 | 0.37298819 | 0.12508925 | 0.00286581 |
| smoking initiation | RA | 58284 | rs11658881 | 0.42511922 | 0.12542375 | 0.0007003 |
| smoking initiation | RA | 58284 | rs11712680 | 0.40119904 | 0.1272322 | 0.00161446 |
| smoking initiation | RA | 58284 | rs117143374 | 0.39460681 | 0.12643465 | 0.00180217 |
| smoking initiation | RA | 58284 | rs12042107 | 0.42943201 | 0.12733368 | 0.00074491 |
| smoking initiation | RA | 58284 | rs12112638 | 0.4106974 | 0.12700478 | 0.00122191 |
| smoking initiation | RA | 58284 | rs12186738 | 0.40892843 | 0.12776057 | 0.00137075 |
| smoking initiation | RA | 58284 | rs12333760 | 0.44349388 | 0.1209472 | 0.00024557 |
| smoking initiation | RA | 58284 | rs12441907 | 0.43165927 | 0.12510395 | 0.00055975 |
| smoking initiation | RA | 58284 | rs12474587 | 0.41654104 | 0.12722849 | 0.00106051 |
| smoking initiation | RA | 58284 | rs12545053 | 0.37776144 | 0.12258793 | 0.00205923 |
| smoking initiation | RA | 58284 | rs12632110 | 0.43339639 | 0.1274838 | 0.0006748 |
| smoking initiation | RA | 58284 | rs13030994 | 0.40461755 | 0.1281907 | 0.00159745 |
| smoking initiation | RA | 58284 | rs13261666 | 0.37347181 | 0.12508334 | 0.00282853 |
| smoking initiation | RA | 58284 | rs134529 | 0.4264061 | 0.12606527 | 0.00071849 |
| smoking initiation | RA | 58284 | rs1385108 | 0.39090271 | 0.12741364 | 0.0021551 |
| smoking initiation | RA | 58284 | rs1435741 | 0.38160513 | 0.12715469 | 0.00268998 |
| smoking initiation | RA | 58284 | rs1445649 | 0.41206769 | 0.12703693 | 0.00117995 |
| smoking initiation | RA | 58284 | rs1869243 | 0.39951201 | 0.12743229 | 0.001718 |
| smoking initiation | RA | 58284 | rs1899896 | 0.39910023 | 0.12729429 | 0.00171704 |
| smoking initiation | RA | 58284 | rs1971318 | 0.40460633 | 0.12750946 | 0.00150796 |
| smoking initiation | RA | 58284 | rs2046850 | 0.41674055 | 0.12670558 | 0.00100527 |
| smoking initiation | RA | 58284 | rs2140114 | 0.40721796 | 0.12768199 | 0.00142611 |
| smoking initiation | RA | 58284 | rs2378662 | 0.40672185 | 0.12761109 | 0.00143658 |
| smoking initiation | RA | 58284 | rs240963 | 0.44169184 | 0.12733501 | 0.00052291 |
| smoking initiation | RA | 58284 | rs2631024 | 0.40742412 | 0.12714161 | 0.00135302 |
| smoking initiation | RA | 58284 | rs266047 | 0.40044214 | 0.12855695 | 0.00184007 |
| smoking initiation | RA | 58284 | rs3001723 | 0.36259327 | 0.12656849 | 0.00417274 |
| smoking initiation | RA | 58284 | rs301805 | 0.39244984 | 0.12689941 | 0.00198408 |
| smoking initiation | RA | 58284 | rs35702515 | 0.41511129 | 0.1267426 | 0.00105576 |
| smoking initiation | RA | 58284 | rs3800227 | 0.41008546 | 0.12697657 | 0.00123957 |
| smoking initiation | RA | 58284 | rs3904512 | 0.4139708 | 0.12703069 | 0.00111875 |
| smoking initiation | RA | 58284 | rs4352629 | 0.43306421 | 0.12775737 | 0.00069959 |
| smoking initiation | RA | 58284 | rs4523689 | 0.40613182 | 0.12807388 | 0.00151876 |
| smoking initiation | RA | 58284 | rs4543592 | 0.39098012 | 0.12695849 | 0.00207286 |
| smoking initiation | RA | 58284 | rs4674993 | 0.39266083 | 0.12666153 | 0.00193469 |
| smoking initiation | RA | 58284 | rs4781977 | 0.40736051 | 0.12795249 | 0.00145413 |
| smoking initiation | RA | 58284 | rs4785836 | 0.39389034 | 0.12619536 | 0.0018007 |
| smoking initiation | RA | 58284 | rs6265 | 0.42177711 | 0.12686747 | 0.00088562 |
| smoking initiation | RA | 58284 | rs6433897 | 0.40684613 | 0.12796919 | 0.00147656 |
| smoking initiation | RA | 58284 | rs66680800 | 0.39859104 | 0.12682205 | 0.00167274 |
| smoking initiation | RA | 58284 | rs6669839 | 0.40104296 | 0.12718616 | 0.00161493 |
| smoking initiation | RA | 58284 | rs6728726 | 0.42578718 | 0.12699635 | 0.00080013 |
| smoking initiation | RA | 58284 | rs6893752 | 0.42819129 | 0.12478357 | 0.00060032 |
| smoking initiation | RA | 58284 | rs7197072 | 0.40986686 | 0.12698603 | 0.0012481 |
| smoking initiation | RA | 58284 | rs7224742 | 0.4382255 | 0.12323582 | 0.00037656 |
| smoking initiation | RA | 58284 | rs72789632 | 0.42867653 | 0.12619016 | 0.00068112 |
| smoking initiation | RA | 58284 | rs7555507 | 0.40722501 | 0.12900035 | 0.00159525 |
| smoking initiation | RA | 58284 | rs76214862 | 0.4040869 | 0.12740185 | 0.00151523 |
| smoking initiation | RA | 58284 | rs7929518 | 0.40072106 | 0.12774632 | 0.00170773 |
| smoking initiation | RA | 58284 | rs7938812 | 0.40371981 | 0.13130481 | 0.00210731 |
| smoking initiation | RA | 58284 | rs7969559 | 0.40126024 | 0.12773774 | 0.0016821 |
| smoking initiation | RA | 58284 | rs9401770 | 0.41709755 | 0.1272433 | 0.00104563 |
| smoking initiation | RA | 58284 | rs962625 | 0.38917153 | 0.12627771 | 0.00205704 |
| smoking initiation | RA | 58284 | rs993700 | 0.39658014 | 0.12735912 | 0.00184649 |
| smoking initiation | RA | 58284 | All | 0.40751389 | 0.12592547 | 0.00121153 |
| smoking initiation | MS | 115803 | rs10001365 | 0.03638737 | 0.08466661 | 0.66736125 |
| smoking initiation | MS | 115803 | rs10114490 | 0.04584436 | 0.08445253 | 0.58723874 |
| smoking initiation | MS | 115803 | rs10233018 | 0.04472253 | 0.08481018 | 0.59796792 |
| smoking initiation | MS | 115803 | rs10260968 | 0.03286101 | 0.08444575 | 0.69717443 |
| smoking initiation | MS | 115803 | rs10279261 | 0.0468935 | 0.08448643 | 0.57886607 |
| smoking initiation | MS | 115803 | rs10498846 | 0.03422563 | 0.08445961 | 0.68530789 |
| smoking initiation | MS | 115803 | rs1050847 | 0.02568133 | 0.08448848 | 0.76115642 |
| smoking initiation | MS | 115803 | rs10905461 | 0.03093761 | 0.08448796 | 0.7142324 |
| smoking initiation | MS | 115803 | rs11057005 | 0.02732321 | 0.08448477 | 0.74638481 |
| smoking initiation | MS | 115803 | rs11078713 | 0.0286516 | 0.08443228 | 0.73435049 |
| smoking initiation | MS | 115803 | rs1154693 | 0.0462223 | 0.08459695 | 0.584803 |
| smoking initiation | MS | 115803 | rs11658881 | 0.04334818 | 0.08444687 | 0.60772827 |
| smoking initiation | MS | 115803 | rs11712680 | 0.04503302 | 0.0844652 | 0.59392649 |
| smoking initiation | MS | 115803 | rs117143374 | 0.03635751 | 0.08438604 | 0.66657927 |
| smoking initiation | MS | 115803 | rs11872397 | 0.04528507 | 0.08450088 | 0.59201909 |
| smoking initiation | MS | 115803 | rs12025237 | 0.05168695 | 0.08452422 | 0.54086555 |
| smoking initiation | MS | 115803 | rs12042107 | 0.02837733 | 0.08444877 | 0.73684817 |
| smoking initiation | MS | 115803 | rs12112638 | 0.04139892 | 0.08453203 | 0.62431618 |
| smoking initiation | MS | 115803 | rs12186738 | 0.03061553 | 0.08464285 | 0.71757439 |
| smoking initiation | MS | 115803 | rs12333760 | 0.05420361 | 0.08451661 | 0.52130395 |
| smoking initiation | MS | 115803 | rs12441907 | 0.03982999 | 0.08468344 | 0.63811226 |
| smoking initiation | MS | 115803 | rs12474587 | 0.0484025 | 0.08482994 | 0.56828249 |
| smoking initiation | MS | 115803 | rs12545053 | 0.04091674 | 0.08443379 | 0.62795906 |
| smoking initiation | MS | 115803 | rs12632110 | 0.04512272 | 0.08454669 | 0.59354791 |
| smoking initiation | MS | 115803 | rs13030994 | 0.0362241 | 0.08550693 | 0.67182885 |
| smoking initiation | MS | 115803 | rs13261666 | 0.04816567 | 0.084825 | 0.57015443 |
| smoking initiation | MS | 115803 | rs134529 | 0.05629885 | 0.08441977 | 0.50484129 |
| smoking initiation | MS | 115803 | rs1385108 | 0.03997255 | 0.08455449 | 0.63639652 |
| smoking initiation | MS | 115803 | rs1435741 | 0.04095083 | 0.08492723 | 0.62967287 |
| smoking initiation | MS | 115803 | rs1445649 | 0.02830834 | 0.08464829 | 0.73806032 |
| smoking initiation | MS | 115803 | rs1869243 | 0.03386614 | 0.08443264 | 0.6883447 |
| smoking initiation | MS | 115803 | rs1899896 | 0.02319683 | 0.08463472 | 0.78402184 |
| smoking initiation | MS | 115803 | rs1971318 | 0.0335527 | 0.08446671 | 0.69119793 |
| smoking initiation | MS | 115803 | rs2046850 | 0.04157657 | 0.08442672 | 0.62239598 |
| smoking initiation | MS | 115803 | rs2140114 | 0.05630116 | 0.08460323 | 0.50574806 |
| smoking initiation | MS | 115803 | rs2378662 | 0.03022107 | 0.08439754 | 0.72028348 |
| smoking initiation | MS | 115803 | rs240963 | 0.03691772 | 0.08502325 | 0.66413788 |
| smoking initiation | MS | 115803 | rs2631024 | 0.04160681 | 0.08444868 | 0.62223339 |
| smoking initiation | MS | 115803 | rs266047 | 0.03627602 | 0.08501922 | 0.66961224 |
| smoking initiation | MS | 115803 | rs3001723 | 0.03469885 | 0.08509277 | 0.68343761 |
| smoking initiation | MS | 115803 | rs301805 | 0.05011486 | 0.08450024 | 0.55313188 |
| smoking initiation | MS | 115803 | rs35702515 | 0.05744441 | 0.08433906 | 0.49580014 |
| smoking initiation | MS | 115803 | rs3800227 | 0.04185532 | 0.08445773 | 0.62019284 |
| smoking initiation | MS | 115803 | rs3801289 | 0.03186218 | 0.08435011 | 0.70562574 |
| smoking initiation | MS | 115803 | rs3904512 | 0.03651561 | 0.08449468 | 0.66562174 |
| smoking initiation | MS | 115803 | rs4044321 | 0.04968561 | 0.08480374 | 0.55794986 |
| smoking initiation | MS | 115803 | rs4236259 | 0.02764873 | 0.08453618 | 0.74361961 |
| smoking initiation | MS | 115803 | rs4352629 | 0.04035212 | 0.08491116 | 0.63462482 |
| smoking initiation | MS | 115803 | rs4523689 | 0.02997966 | 0.08445463 | 0.72260498 |
| smoking initiation | MS | 115803 | rs4543592 | 0.0314481 | 0.08452595 | 0.70985371 |
| smoking initiation | MS | 115803 | rs4674993 | 0.02905224 | 0.08444472 | 0.73081731 |
| smoking initiation | MS | 115803 | rs4781977 | 0.0380247 | 0.08440151 | 0.65233443 |
| smoking initiation | MS | 115803 | rs4785836 | 0.04300139 | 0.08442775 | 0.61052257 |
| smoking initiation | MS | 115803 | rs6265 | 0.03580722 | 0.08471073 | 0.67251424 |
| smoking initiation | MS | 115803 | rs6433897 | 0.05696462 | 0.08443438 | 0.4998909 |
| smoking initiation | MS | 115803 | rs66680800 | 0.03604956 | 0.0844406 | 0.66943607 |
| smoking initiation | MS | 115803 | rs6669839 | 0.04171566 | 0.08445557 | 0.62135124 |
| smoking initiation | MS | 115803 | rs6728726 | 0.03806592 | 0.08482352 | 0.65360041 |
| smoking initiation | MS | 115803 | rs6893752 | 0.02895178 | 0.08450049 | 0.73188241 |
| smoking initiation | MS | 115803 | rs7197072 | 0.02138611 | 0.08446691 | 0.80012204 |
| smoking initiation | MS | 115803 | rs7224742 | 0.04028785 | 0.08447327 | 0.63341186 |
| smoking initiation | MS | 115803 | rs72789632 | 0.05166759 | 0.08455085 | 0.54114459 |
| smoking initiation | MS | 115803 | rs7322872 | 0.03848083 | 0.08430076 | 0.64805147 |
| smoking initiation | MS | 115803 | rs7555507 | 0.03692928 | 0.08464731 | 0.66263912 |
| smoking initiation | MS | 115803 | rs76214862 | 0.02936664 | 0.08439846 | 0.72787602 |
| smoking initiation | MS | 115803 | rs78411160 | 0.03141087 | 0.08445435 | 0.70994704 |
| smoking initiation | MS | 115803 | rs7929518 | 0.05657284 | 0.08447203 | 0.50303455 |
| smoking initiation | MS | 115803 | rs7938812 | 0.04784405 | 0.08605479 | 0.57823008 |
| smoking initiation | MS | 115803 | rs7969559 | 0.03219042 | 0.08455282 | 0.7034157 |
| smoking initiation | MS | 115803 | rs9401770 | 0.02855129 | 0.08471207 | 0.73608735 |
| smoking initiation | MS | 115803 | rs962625 | 0.03664828 | 0.08447882 | 0.66442197 |
| smoking initiation | MS | 115803 | rs993700 | 0.04548327 | 0.08454321 | 0.59058503 |
| smoking initiation | MS | 115803 | All | 0.03902101 | 0.083997 | 0.64225201 |

Abbreviations: MS, multiple sclerosis; RA, rheumatoid arthritis; beta, estimate coefficient; se, standard error of coefficient estimate.

## Supplementary Table S18 Effect estimates by IVW method in multivariable MR analyses.

|  | Exposure | Outcome | b | se | OR (95%CI) | P-value |
| --- | --- | --- | --- | --- | --- | --- |
| MCP+BMI | MCP | MS | 0.59142008 | 0.24824868 | 1.80 (1.11, 2.94) | 0.017 |
| MCP+BMI | BMI | MS | -0.5147711 | 0.58556656 | 0.59 (0.19, 1.88) | 0.379 |
| MCP+BMI | MCP | RA | -0.6565576 | 0.7774729 | 2.21 (1.05,4.68) | 0.037 |
| MCP+BMI | BMI | RA | 0.79403518 | 0.38197767 | 0.52 (0.11,2.38) | 0.398 |
| MCP+Smoking | MCP | MS | 0.37194018 | 0.30330519 | 1.45 (0.80, 2.63) | 0.220 |
| MCP+Smoking | Smoking | MS | 0.14098589 | 0.43718516 | 1.15 (0.49, 2.71) | 0.747 |
| MCP+Smoking | MCP | RA | 0.39375046 | 0.32036307 | 1.48 (0.79, 2.78) | 0.219 |
| MCP+Smoking | Smoking | RA | 0.35433277 | 0.5007006 | 1.43 (0.53, 3.80) | 0.479 |

Abbreviations: MCP, multisite chronic pain; BMI, body mass index; MS, multiple sclerosis; RA, rheumatoid arthritis; OR, odds ratio. beta, estimate coefficient.

## Supplementary Table S19 Effect estimates and Heterogeneity test by MR egger method in multivariable MR analyses.

| **Model** | **Exposure** | **Outcome: MS** | | | | **Outcome: RA** | | | |
| --- | --- | --- | --- | --- | --- | --- | --- | --- | --- |
|  |  | **beta (95%CI)** | **P** | **Q** | **Q_P** | **beta (95%CI)** | **P** | **Q** | **Q_P** |
| MCP+BMI | MCP | 0.60  (0.09, 1.11) | 0.02 | 19.6 | 0.49 | 0.003  (-0.01, 0.02) | 0.69 | 22.1 | 0.18 |
| MCP+BMI | BMI | -0.662  (-2.48,1.16) | 0.48 |  |  | -0.998  (-3.27,1.28） | 0.39 |  |  |
| MCP+Smoking | MCP | 0.48  (-1.93,2.88) | 0.70 | 37.1 | 0.12 | 0.10  (-2.23, 2.43) | 0.93 | 26.8 | 0.31 |
| MCP+Smoking | Smoking | 0.15  (-0.75,1.06) | 0.74 |  |  | 0.31  (-0.74, 1.37) | 0.56 |  |  |

Abbreviations: MCP, multisite chronic pain; BMI, body mass index; MS, multiple sclerosis; RA, rheumatoid arthritis; OR, odds ratio. beta, estimate coefficient.

## Supplementary Table S20 MR results for CWP and AIDs.

| Exposure | Outcome | Method | N  SNP | beta | se | *P*-value |
| --- | --- | --- | --- | --- | --- | --- |
| CWP | SLE | IVW | 2 | -9.4290932 | 7.50245617 | 0.20882576 |
| CWP | ALS | IVW | 3 | -4.7450804 | 3.88419921 | 0.22184504 |
| CWP | ALS | MR Egger | 3 | 29.406797 | 22.411846 | 0.41457963 |
| CWP | ALS | Simple mode | 3 | -8.5860844 | 5.56175935 | 0.26262936 |
| CWP | ALS | Weighted median | 3 | -7.3357827 | 4.18618863 | 0.07970892 |
| CWP | ALS | Weighted mode | 3 | -8.5860844 | 5.22855402 | 0.24226259 |
| CWP | T1D | MR Egger | 3 | 41.6747874 | 37.9353877 | 0.47011905 |
| CWP | T1D | Weighted median | 3 | -0.8414507 | 6.28861772 | 0.8935565 |
| CWP | T1D | IVW | 3 | -1.8064838 | 5.15871542 | 0.72620291 |
| CWP | T1D | Simple mode | 3 | 0.45465585 | 7.69708796 | 0.95826861 |
| CWP | T1D | Weighted mode | 3 | 0.63643876 | 8.11606713 | 0.94463576 |
| CWP | IBD | Wald ratio | 1 | -14.029882 | 3.82455882 | 0.00024411 |
| CWP | RA | IVW | 2 | -1.7083146 | 6.20580773 | 0.78310362 |
| CWP | MS | IVW | 2 | 3.29763673 | 4.38113868 | 0.45163657 |

Abbreviations: CWP, chronic widespread pain; beta, estimate coefficient; se, standard error of coefficient estimate; ALS, amyotrophic lateral sclerosis; IBD, inflammatory bowel disease; MS, multiple sclerosis; RA, rheumatoid arthritis; SLE, systemic lupus erythematosus; T1D, Type 1 diabetes; IVW, Inverse variance weighted.

## Supplementary Table S21 Heterogeneity for CWP and AIDs.

| Outcome | Exposure | Method | Q | Q_df | *P*-value |
| --- | --- | --- | --- | --- | --- |
| SLE | CWP | IVW | 0.03243503 | 1 | 0.85707619 |
| ALS | CWP | MR Egger | 4.1220439 | 11 | 0.96620988 |
| ALS | CWP | IVW | 13.3624621 | 12 | 0.34325495 |
| T1D | CWP | MR Egger | 0.00245235 | 1 | 0.96050397 |
| T1D | CWP | IVW | 1.34096278 | 2 | 0.51146231 |
| RA | CWP | IVW | 0.2080594 | 1 | 0.6482927 |
| MS | CWP | IVW | 0.3226675 | 1 | 0.57000897 |

Abbreviations: CWP, chronic widespread pain; ALS, amyotrophic lateral sclerosis; MS, multiple sclerosis; RA, rheumatoid arthritis; SLE, systemic lupus erythematosus; T1D, Type 1 diabetes; IVW, Inverse variance weighted.

## Supplementary Table S22 Pleiotropy for chronic widespread pain and AIDs.

| Exposure | Outcome | egger_intercept | se | *P*-value |
| --- | --- | --- | --- | --- |
| CWP | SLE | NA | NA | NA |
| CWP | ALS | -0.1113964 | 0.0366459 | 0.0112505 |
| CWP | T1D | -0.1442802 | 0.12470848 | 0.45376069 |
| CWP | RA | NA | NA | NA |
| CWP | MS | NA | NA | NA |

Abbreviations: CWP, chronic widespread pain; se, standard error of coefficient estimate; ALS, amyotrophic lateral sclerosis; MS, multiple sclerosis; RA, rheumatoid arthritis; SLE, systemic lupus erythematosus; T1D, Type 1 diabetes.
